# Cross-Informant Agreement and Discrepancies in ADHD Assessment During Adolescence: A Systematic Review and Meta-Analysis

**DOI:** 10.3390/children13091176

**Published:** 2026-09-01

**Authors:** Georgios Giannakopoulos, Afroditi Prassou

**Affiliations:** 1 Department of Child and Adolescent Psychiatry, School of Medicine, National and Kapodistrian University of Athens, “Aghia Sophia” Children’s Hospital, 115 27 Athens, Greece; 2 4th Directorate of Secondary Education of Athens, 171 21 Athens, Greece; aprassou@sch.gr

**Keywords:** ADHD, adolescence, informant agreement, informant discrepancies, parent report, teacher report, self-report, meta-analysis

## Abstract

**Highlights:**

**What are the main findings?**

- Cross-informant agreement for adolescent attention-deficit/hyperactivity disorder symptoms was positive but modest and highly heterogeneous across caregiver, adolescent and teacher reports.
- Caregiver–adolescent pooled correlations averaged *r* = 0.33 for inattention and *r* = 0.32 for hyperactivity/impulsivity, with prediction intervals crossing zero; caregiver–teacher estimates were similar in magnitude, highly heterogeneous, and imprecise.

**What are the implications of the main findings?**

- Discrepant ratings should be treated as source- and context-specific clinical information rather than resolved by selecting one informant as universally more accurate.
- Assessment and research should document informant identity, setting, independence, timing and both dimensional and categorical agreement using age-stratified results.

**Abstract:**

Background/Objectives: Assessment of attention-deficit/hyperactivity disorder (ADHD) requires information across settings, but reports from adolescents, caregivers, teachers, and clinicians are often only partly concordant. This review synthesized cross-informant agreement and discrepancies in ADHD assessment among adolescents aged 10–19 years. Methods: MEDLINE, CINAHL, ERIC and Academic Search Complete were searched through EBSCO, together with PubMed and Scopus, on 19 July 2026. Eligible primary quantitative studies compared at least two independent informants assessing the same adolescent ADHD domain. Methodological quality was appraised with an adapted 11-item QAREL framework. Pearson correlations were pooled after Fisher *z* transformation using restricted maximum-likelihood random-effects models and Hartung–Knapp–Sidik–Jonkman confidence intervals. Results: The searches identified 1447 records; 30 reports (282 outcomes) were included. Caregiver–adolescent pooled *r* was 0.33 for inattention (*k* = 7, 95% CI 0.19–0.46; *I*^2^ = 88.1%; 95% prediction interval −0.07 to 0.64) and 0.32 for hyperactivity/impulsivity (*k* = 7, 95% CI 0.12–0.50; *I*^2^ = 94.8%; prediction interval −0.28 to 0.74). Despite the positive pooled point estimates, both prediction intervals were wide and crossed zero. Caregiver–teacher estimates were *r* = 0.29 for inattention (*k* = 4, 95% CI −0.02 to 0.55; *I*^2^ = 94.1%) and *r* = 0.27 for hyperactivity/impulsivity (*k* = 4, 95% CI −0.03 to 0.52; *I*^2^ = 83.0%); both confidence intervals crossed zero. Diagnostic agreement was generally low and sensitive to impairment and consensus rules. Conclusions: Informant reports were positively related on average, but the magnitude of agreement was highly heterogeneous and imprecisely estimated. Wide intervals, including caregiver–adolescent prediction intervals crossing zero and caregiver–teacher confidence intervals crossing zero, indicate that little or no agreement remains plausible in some comparable settings. Reports should therefore not be treated as clinically interchangeable, and pooled estimates should not be interpreted as universal levels of agreement.

## 1. Introduction

Assessment of attention-deficit/hyperactivity disorder (ADHD) is intrinsically multi-contextual. Symptoms must be evaluated alongside impairment and their expression across settings, which makes reports from adolescents, caregivers, teachers and clinicians central to diagnostic formulation and treatment planning [1,2,3,4]. These sources do not observe the same behavior under the same demands. Caregivers mainly observe routines and relationships at home, teachers observe performance under structured academic and peer demands, and adolescents have direct access to effort, distraction and internal experiences that may not be visible to adults [1,4,5,6].

Cross-informant differences are common throughout child and adolescent mental health assessment. Classic and more recent work indicates that agreement is generally lower when informants observe young people in different settings and that discrepancies may reflect context-specific behavior, different thresholds for concern, distinct relationships with the young person, and characteristics of the assessment method [7,8]. Disagreement should therefore not automatically be interpreted as evidence that one informant is inaccurate. The value of a multi-informant assessment lies not in expecting different sources to reproduce one another, but in using their convergence and divergence to clarify where, when, and to whom difficulties are apparent [8,9]. Privileging a single report may overlook clinically important difficulties, whereas combining reports without examining their differences may obscure meaningful variation across settings [8,9,10].

Adolescence makes this issue particularly consequential. Parental observation becomes less complete, school-based information may be distributed across several teachers, and adolescents assume a more prominent role in describing their own functioning [1,5,6]. Adolescents may be better positioned to report the effort required to sustain attention or regulate behavior, while caregivers and teachers may be better placed to evaluate observable behavior, consistency over time, and interference with everyday functioning [11,12,13,14,15]. Informants may also apply different thresholds when deciding whether a behavior is problematic, depending on their expectations, opportunities for observation, and relationship with the adolescent [16,17,18]. Cross-informant discrepancies during adolescence may therefore contain information about the contextual distribution and functional significance of ADHD symptoms rather than merely representing measurement noise [8,13,14,19,20,21].

Agreement is also not a unitary construct. Correlations between rating scales indicate whether two informants rank adolescents in a similar order, but they do not establish agreement in absolute symptom levels or diagnostic classification [10,22,23]. Conversely, paired mean differences can identify a systematic tendency for one informant to report more symptoms than another without showing whether the two reports correspond at the individual level. Intraclass correlations, kappa coefficients, agreement proportions, and latent-variable models address related but distinct aspects of concordance [22,24,25,26,27]. These outcomes should not be treated as interchangeable, particularly because clinical decisions depend on symptom thresholds, impairment, and cross-setting evidence rather than solely on relative rank ordering [8,28,29].

Cross-informant disagreement, clinical non-interchangeability, and psychometric non-equivalence are distinct concepts. In this review, cross-informant disagreement refers to observed differences in symptom scores, classifications, or other agreement outcomes across reporters. Clinical non-interchangeability means that reports from different informants should not be assumed to provide the same information or to be substitutable in clinical formulation. Psychometric non-equivalence refers more narrowly to evidence that items or latent constructs function differently across informants, usually evaluated through measurement-invariance or related analyses [30]. Modest correlations or discrepant scores alone do not demonstrate psychometric non-equivalence.

Adjacent reviews have examined the validity of teacher rating scales against diagnostic interviews or classroom observations [31] and the measurement invariance of ADHD symptom criteria across parent and teacher reports [30]. These questions concern criterion validity and psychometric equivalence, respectively, and are related to but distinct from direct cross-informant agreement. Neither review directly synthesized cross-informant agreement across the range of informant pairs relevant to adolescent assessment. The pertinent primary literature includes parent–adolescent, parent–teacher, teacher–adolescent, teacher–teacher, and clinician or consensus comparisons and employs markedly different definitions and indices of agreement [8,9,30,31]. An adolescence-specific synthesis is therefore needed to determine whether observed discrepancies represent a broadly consistent pattern or are mainly attributable to particular informant pairs, instruments, outcome definitions, or diagnostic rules.

This review aimed to synthesize the magnitude, direction, and methodological determinants of cross-informant agreement and discrepancies in ADHD assessment among adolescents aged 10–19 years. The primary questions concerned agreement between caregivers and adolescents, and between caregivers and teachers, for the core domains of inattention and hyperactivity/impulsivity. Secondary questions concerned diagnostic and threshold agreement, teacher–adolescent and teacher–teacher agreement, comparisons with clinicians or consensus diagnoses, systematic mean differences, assessment format effects, and proposed moderators of disagreement.

## 2. Materials and Methods

### 2.1. Protocol and Reporting Framework

This systematic review and exploratory meta-analysis was conducted according to the Preferred Reporting Items for Systematic Reviews and Meta-Analyses (PRISMA) 2020 statement [32]. The completed PRISMA 2020 checklist is provided in Table S1. The review was registered in the Open Science Framework (OSF) Registries on 22 July 2026 (https://doi.org/10.17605/OSF.IO/49H5R (accessed on 20 August 2026)). Database searching and initial study retrieval had already been completed before registration. The review question, age range, broad eligibility framework, information sources, search strings, and search date had therefore already been established and are not presented as prospectively registered decisions.

Registration occurred before formal data extraction, methodological quality appraisal, effect grouping, and quantitative synthesis. The registered synthesis framework specified the separate treatment of conceptually different agreement and discrepancy metrics, domain-specific analyses, a minimum of three independent studies for quantitative pooling, a random-effects synthesis, and planned robustness checks. Thus, the minimum threshold of three independent studies for pooling was specified in the registered plan, whereas the designation of the four specific domain-by-informant Pearson correlation models as the primary quantitative syntheses was made after registration.

After registration, but before interpretation of the pooled results, several operational decisions were documented in the review workbook. These included preference for DSM or core-domain scales over broader content scales, selection of parallel rating-scale effects where multiple formats were available, implementation of the dependence rule for non-overlapping age strata, specification of restricted maximum likelihood estimation and Hartung–Knapp–Sidik–Jonkman confidence intervals, and designation of four domain-specific Pearson-correlation models as the primary quantitative syntheses.

### 2.2. Eligibility Criteria

Eligibility was structured around the population, phenomenon of interest, comparison and outcome. Studies were eligible when they met all of the following criteria:

- Participants were aged 10–19 years, or the report presented a result that was separable for this age range.
- At least two independent informants assessed the same adolescent, including adolescents, parents or caregivers, teachers, clinicians, consensus teams, or two raters of the same eligible type. For synthesis-level reporting, “caregiver” is used throughout as an umbrella term encompassing parents, mothers, fathers, primary caregivers, caretakers, and legal guardians. Source-specific informant labels are retained when individual studies are described.
- The compared construct was an ADHD diagnosis, ADHD symptom total, inattention, hyperactivity/impulsivity, or a clearly ADHD-specific scale or classification.
- The report presented a quantitative cross-informant agreement or discrepancy analysis, such as Pearson correlation, ICC, kappa, agreement proportion, diagnostic concordance, paired classification, paired mean difference, or a compatible model-based estimate.
- The study was primary quantitative research. Baseline and secondary analyses of trials or cohorts were eligible when the relevant agreement data were available.

Studies were excluded when the eligible adolescent component of a mixed-age sample could not be separated; only one informant was used; the comparison concerned two instruments completed by the same person, chart coders, administrative records or treatment-response ratings; the outcome was not ADHD-specific; no direct agreement or discrepancy analysis was reported; or the record was a review, protocol, commentary, case report or other non-primary publication. No publication-year or language restriction was applied.

### 2.3. Information Sources and Search Strategy

Searches were completed on 19 July 2026 in MEDLINE, CINAHL with Full Text, ERIC and Academic Search Complete through the EBSCO interface; PubMed through the PubMed interface; and Scopus through the Scopus interface. APA PsycInfo was not available through the accessible institutional interface and could therefore not be searched directly. To broaden coverage beyond biomedical indexing, the search included Scopus and Academic Search Complete in addition to MEDLINE, PubMed, CINAHL and ERIC, using broad terms for informants, agreement, discrepancy and reliability. No separate formal backward- or forward-citation-chasing stage was conducted. The strategy combined free-text terms for ADHD, adolescence, informants or reporters, and agreement, concordance, discrepancy or inter-rater reliability; PubMed Medical Subject Headings (MeSH) terms were added where applicable. Database syntax and field tags were adapted to each interface; the complete database-specific search strategies and yields are presented in Table S2.

The database yields were MEDLINE 243, CINAHL 75, ERIC 29, Academic Search Complete 165, PubMed 531 and Scopus 404, for a gross total of 1447 records. Bibliographic records were exported in RIS or NBIB format. Full-text retrieval used publisher platforms, DOI links, institutional access, PubMed Central and university or author repositories; authors were approached when an eligible report could not otherwise be obtained.

### 2.4. Record Management and Study Selection

Records from all sources were merged into a master corpus. Deduplication used persistent identifiers where available and normalized title matching, followed by manual review of potential collisions. The resulting 796 unique records were initially screened at title and abstract level by one reviewer. Uncertain records were retained for full-text assessment rather than excluded.

During peer-review revision, the second reviewer first completed a calibration exercise on 50 title-and-abstract records to ensure consistent application of the eligibility criteria. She then independently rescreened all 796 unique records without access to the original screening decisions. The second reviewer also independently reassessed all 64 retrieved full-text reports without access to the original final decisions or exclusion reasons. Disagreements were resolved through discussion using the registered eligibility criteria and exclusion hierarchy.

Four records that had initially been excluded at title-and-abstract level were advanced and subsequently met the eligibility criteria. The final workflow therefore comprised 68 reports sought for retrieval, 64 reports assessed in full text, four reports not retrieved, 34 full-text exclusions, and 30 included reports.

### 2.5. Data Extraction

A standardized extraction form was piloted on five reports and then applied to the remaining included reports. Extraction captured bibliographic information; country, setting and design; recruitment and sample characteristics, including the eligible age range or stratum and mean age where reported; informant pairs; instruments, administration procedures and scoring; the exact outcome definition; metric, estimate, uncertainty and analysis sample; time point; effect direction; and source location in the report. Results were retained at the most granular level needed to distinguish symptom domain, informant pair, age stratum, assessment method and case definition. One reviewer performed the initial extraction and a same-reviewer second pass against the full reports. Four additional eligible reports identified during revision were extracted using the same coding rules. During revision, the second reviewer independently verified all 22 study-domain effect entries contributing to the four final primary meta-analyses against the source reports. These entries originated from nine unique reports. Verification covered the analysis-specific sample size, informant pair, ADHD domain, reported Pearson correlation, source page or table, primary-effect selection, and any within-study combination or transformation. Disagreements were resolved by returning to the source report and applying the prespecified extraction and effect-selection rules. The remaining result-level rows used in the structured narrative synthesis were not independently extracted in duplicate. Missing values were left as missing and were not imputed. The final dataset comprised 30 included reports and 282 result-level rows.

### 2.6. Methodological Quality Appraisal

Methodological quality was assessed at report level using an adapted 11-item Quality Appraisal of Reliability Studies (QAREL) framework [33]. QAREL was selected because it was developed specifically for studies of diagnostic reliability and agreement and therefore addresses methodological features that are directly relevant to cross-informant assessment, including the suitability of the participant and rater spectra, independence of ratings, potential information or order effects, temporal proximity of assessments, and the appropriate application and interpretation of agreement statistics.

The framework was adapted operationally to the cross-informant ADHD literature rather than used as a generic risk-of-bias scale. Q1 and Q2 addressed the suitability and representativeness of the adolescent sample and the informants being compared. Q3–Q8 were interpreted in relation to independence of informant completion, exposure to another informant’s ratings or other clinical cues, and possible order effects. Q9 addressed whether the compared assessments were sufficiently close in time for meaningful agreement to be expected. Q10 evaluated whether the reported agreement statistic was correctly applied and interpreted, including whether a correlation was described more broadly than its rank-order meaning justified. Q11 evaluated the appropriateness of the statistical analysis for the stated agreement question. Items that were structurally irrelevant to a particular assessment design were coded Not applicable rather than treated as methodological failures.

Each item was judged Yes, No, Unclear, or Not applicable. Overall judgments of Low concern, Some concerns, or High concern were qualitative and reflected the likely methodological impact of the identified issues rather than a summed item score. Reports were judged High concern when one or more limitations could materially affect the interpretation of the agreement estimate, whereas Some concerns generally reflected incomplete reporting or limitations of less certain impact. No numerical QAREL score or cutoff was calculated.

The initial appraisal was completed by one reviewer. During peer-review revision, the second reviewer independently repeated the item-level QAREL appraisal for all 30 included reports using the same operational interpretation guide and without access to the original overall judgments. Disagreements were resolved through discussion and re-examination of the source reports before the final report-level judgments were assigned.

### 2.7. Outcome Grouping and Effect Selection

After data extraction and effect grouping, but before interpretation of the pooled results, the quantitative synthesis was operationalized as four primary Pearson-correlation models: caregiver–adolescent agreement for inattention and hyperactivity/impulsivity, and caregiver–teacher agreement for the same two domains. The registered synthesis framework had specified domain-specific analyses, separate treatment of conceptually different agreement metrics, and a minimum of three independent studies for quantitative pooling; designation of these four models as the primary quantitative syntheses was a post-registration operational decision. One domain-aligned effect was selected per independent study. DSM or core symptom-domain scales were preferred to broader content scales. When a study reported multiple eligible assessment formats, the parallel rating-scale result was primary and the alternative format was retained for sensitivity analysis. Non-overlapping age strata from the same report were combined on the Fisher-*z* scale before entering the model.

Kappa coefficients, ICCs, agreement proportions, mean discrepancies and latent-variable estimates were not converted to Pearson correlations because they quantify different properties and often lacked the information required for defensible variance estimation. These outcomes were synthesized in structured narrative form, organized by informant pair, domain, assessment method and diagnostic rule.

### 2.8. Statistical Analysis

Pearson correlations were transformed to Fisher *z* values, with sampling variance calculated as 1/(*n*−3). Inverse-variance random-effects models were fitted using restricted maximum likelihood to estimate between-study variance. Because each model contained few studies, uncertainty around pooled effects was calculated with Hartung–Knapp–Sidik–Jonkman *t* intervals using *k*−1 degrees of freedom. Summary estimates and confidence limits were back-transformed to Pearson *r*. Statistical heterogeneity was described using Cochran’s *Q*, between-study variance (τ^2^) on the Fisher-*z* scale, and the conventional *Q*-based *I*^2^ statistic, calculated as max [0, (*Q* − *df*)/*Q*] × 100. Ninety-five percent prediction intervals were calculated when at least five studies were available.

Formal subgroup analyses and meta-regression were not undertaken because the four primary models contained only four or seven studies and no candidate moderator was reported in a sufficiently consistent form across contributing studies. Age was generally available as broad, overlapping ranges, whereas mean age, sex distribution, parental ADHD, medication status, family characteristics, school context, and recruitment setting were not available in a form that could be harmonized across the studies in each meta-analysis. Potential sources of heterogeneity were therefore examined descriptively through study characteristic comparisons and through QAREL-restricted, alternative-measure, and leave-one-out sensitivity analyses.

Robustness checks comprised fixed-effect models; exclusion of reports judged High concern on QAREL when at least three studies remained; substitution of alternative eligible instruments or formats; and leave-one-out analyses. Funnel plots and regression-based tests of asymmetry were not performed because every meta-analysis included fewer than 10 studies. Meta-analyses were conducted in IBM SPSS Statistics, version 31.0 (IBM Corp., Armonk, NY, USA), using the META ES CONTINUOUS procedure with a random-effects model, restricted maximum-likelihood estimation, and the untruncated Knapp–Hartung standard-error adjustment (ADJUSTSE = KNAPP_HARTUNG). In SPSS this option is distinct from TRUNCATED_KNAPP_HARTUNG and therefore does not impose a lower bound of 1 on the variance adjustment. To verify the implementation, the four primary models were independently recalculated on the Fisher-*z* scale using REML weights, the standard untruncated Hartung–Knapp variance estimator, and *t* critical values with *k*−1 degrees of freedom. The independently calculated pooled estimates and confidence limits reproduced the SPSS results to the reported precision. The analysis syntax, input data and output files were retained for reproducibility.

## 3. Results

### 3.1. Study Selection

**Figure 1:**
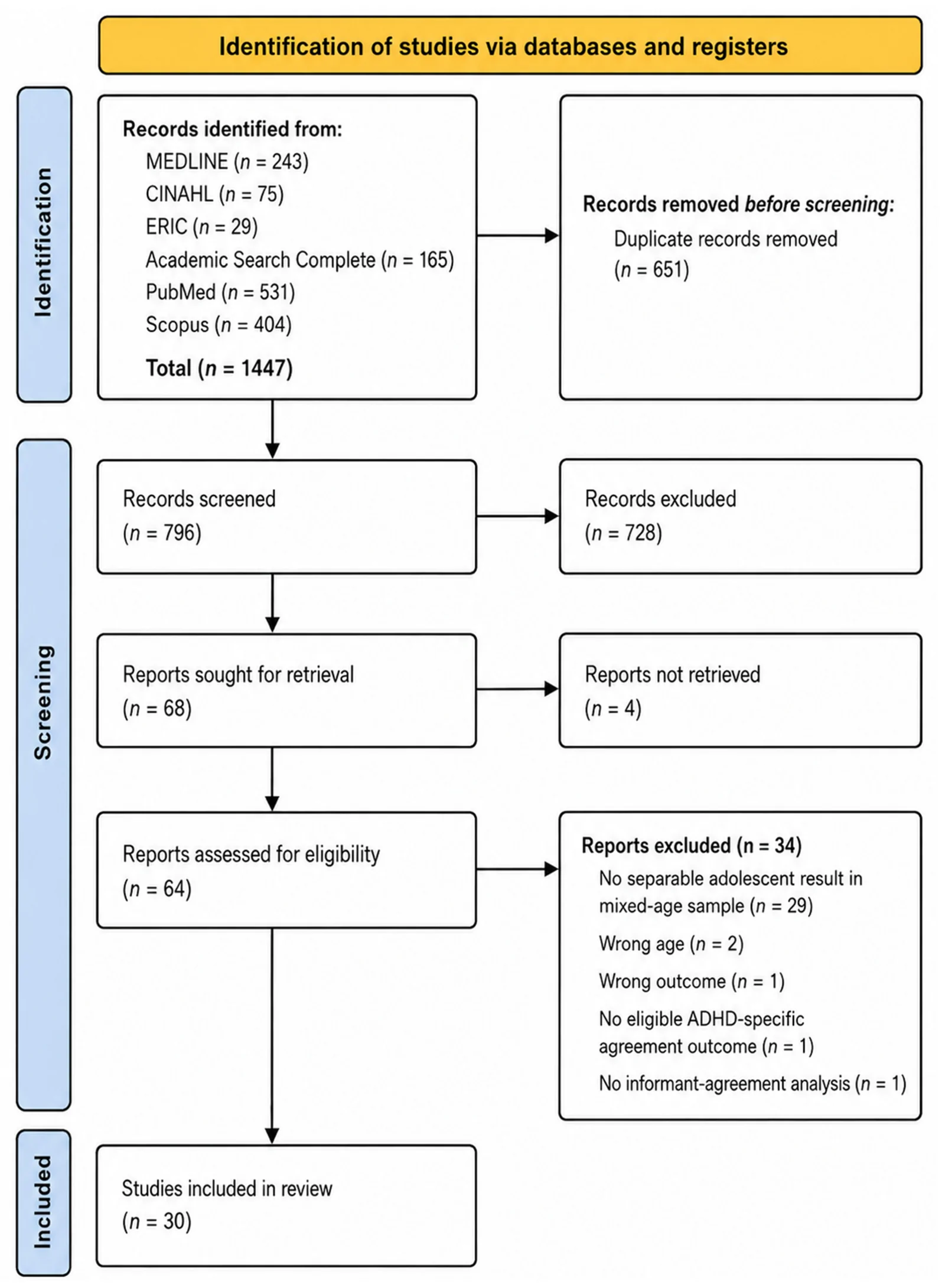
PRISMA 2020 flow diagram for study selection.

The searches yielded 1447 records. After removal of 651 duplicates, 796 unique records were screened. Following independent rescreening during revision, 68 reports were sought for full-text assessment; 64 were retrieved and four were not retrieved. Thirty reports met the review criteria and 34 were excluded after full-text assessment. The most common reason for full-text exclusion was the absence of a separable adolescent result in a mixed-age sample (*n* = 29), followed by wrong age (*n* = 2), wrong outcome (*n* = 1), no eligible ADHD-specific agreement outcome (*n* = 1), and no informant agreement analysis (*n* = 1). The final evidence set comprised 30 reports, all of which were extracted (Figure 1).

### 3.2. Study Characteristics

Included reports were published from 1996 to 2025 and represented clinical, community, school, epidemiologic and follow-up samples across 13 countries or national settings. Report-level sample sizes ranged from 35 to 10,476 participants, with several reports contributing multiple cohorts, age strata or repeated assessments. Informant comparisons included adolescent–caregiver, caregiver–teacher, adolescent–teacher, teacher–teacher, and comparisons with clinicians or consensus diagnoses. Instruments included DSM-based symptom scales, Conners forms, Vanderbilt and SNAP-IV ratings, structured or semi-structured diagnostic interviews, broadband behavior measures, and multi-trait multi-method or latent-class models. The included reports are summarized in Table S3. No language restriction was applied. Of the 30 included reports, 29 were published in English and one was published in Spanish. No report was excluded solely on the basis of publication language.

Age coverage was heterogeneous and is reported in detail in Table S3. The eligible evidence was concentrated between ages 10 and 18; no included report provided an age-19-specific agreement estimate. Among the seven caregiver–adolescent studies contributing to the primary correlation models [34,35,36,37,38,39,40], age ranges overlapped substantially, spanning 11–18 years overall, and mean ages ranged from 12.78 to 16.24 years. Among the four studies contributing to each caregiver–teacher model [3,37,39,41], mean age was reported for three studies (12.78, 15.00, and 16.24 years), while Montiel-Nava and Peña [41] reported separate 12–14 and 15–17-year strata. These data make the developmental coverage of the evidence visible but do not support a reliable age-gradient analysis.

The 282 extracted outcomes comprised Pearson correlations, ICCs, kappa coefficients, exact or positive-case agreement proportions, paired classifications, mean discrepancies, regression-based discrepancy effects, latent-variable estimates, and model-based class proportions. This variation supported quantitative pooling only for the four Pearson-correlation groups.

### 3.3. Methodological Quality

All 30 included reports were assessed with QAREL: three were judged “Low concern”, 15 “Some concerns” and 12 “High concern”. Recurrent concerns were insufficient reporting of masking or independent completion, fixed interview order or the same interviewer having access to both reports, nonparallel instruments, age-stratum uncertainty, and use of correlation alone to describe “agreement.” Several studies had otherwise appropriate same-period measurements and suitable participant and rater spectra, but failed to describe procedures that would prevent influence between informants. Report-level methodological quality judgments are presented in Table S4. Because 27 of the 30 included reports had at least some methodological concern, the primary estimates were interpreted alongside the QAREL-restricted sensitivity analyses rather than as equally robust across all contributing studies. The influence of methodological quality on the quantitative findings is reported in Section 3.7.

### 3.4. Caregiver–Adolescent Agreement

**Figure 2:**
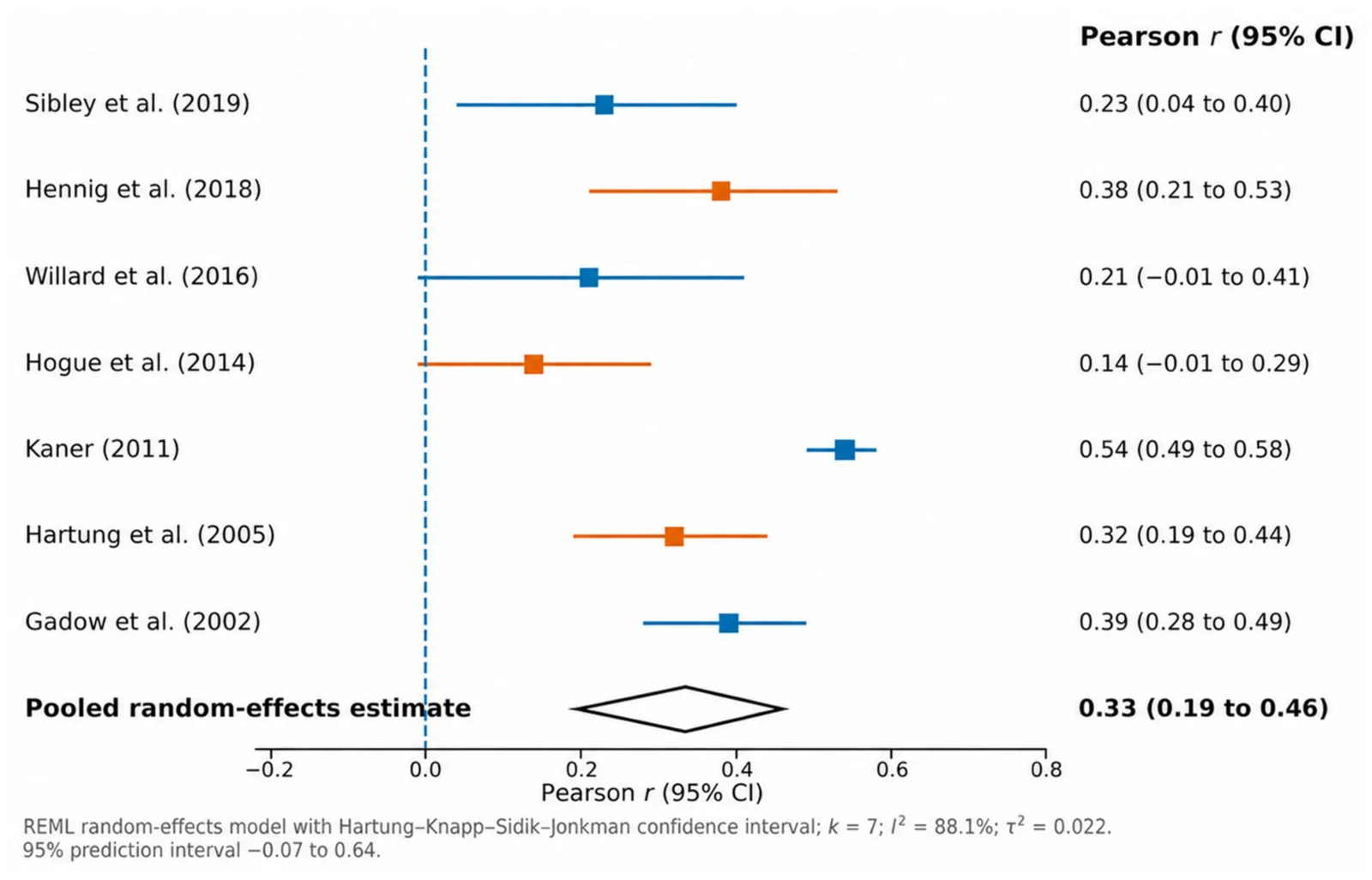
Forest plot of caregiver–adolescent agreement for inattention [34,35,36,37,38,39,40]. Note: Individual confidence intervals were calculated by normal approximation on the Fisher *z* scale; the pooled confidence interval used the Hartung–Knapp–Sidik–Jonkman method. Effect sizes and confidence intervals were back-transformed to Pearson *r* for presentation.

**Figure 3:**
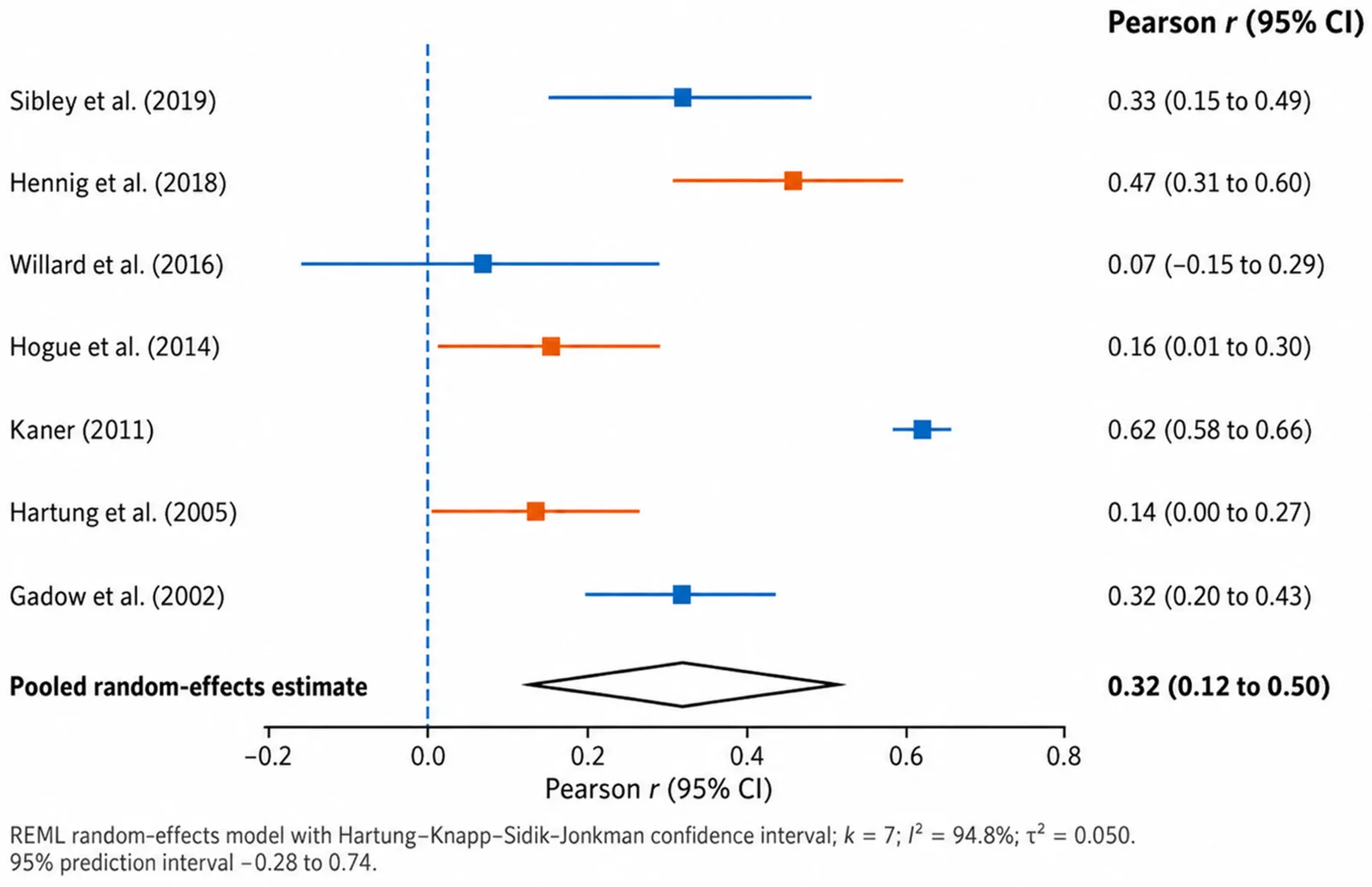
Forest plot of caregiver–adolescent agreement for hyperactivity/impulsivity [34,35,36,37,38,39,40]. Note: Individual confidence intervals were calculated by normal approximation on the Fisher *z* scale; the pooled confidence interval used the Hartung–Knapp–Sidik–Jonkman method. Effect sizes and confidence intervals were back-transformed to Pearson *r* for presentation.

Seven studies contributed to each caregiver–adolescent meta-analysis [34,35,36,37,38,39,40]. For inattention, the random-effects pooled correlation was *r* = 0.33 (95% CI 0.19–0.46, *p* = 0.001). Heterogeneity remained high, *Q*(6) = 50.33, *p* < 0.001, *I*^2^ = 88.1%, τ^2^ = 0.022, and the 95% prediction interval extended from −0.07 to 0.64. For hyperactivity/impulsivity, the pooled correlation was *r* = 0.32 (95% CI 0.12–0.50, *p* = 0.009), with *Q*(6) = 115.25, *p* < 0.001, *I*^2^ = 94.8%, τ^2^ = 0.050 and a prediction interval from −0.28 to 0.74. Thus, the pooled estimates represent average associations across highly heterogeneous studies rather than stable levels of agreement. Both prediction intervals crossed zero and extended to substantially positive correlations, indicating that a future comparable study could plausibly observe little, no, or much stronger correspondence. Summary estimates are presented in Table 1 and study-level estimates in Figure 2 and Figure 3. Study-level inputs for all primary meta-analyses are provided in Table S5.

Diagnostic and threshold outcomes did not support a common meta-analysis. Kappa estimates for caregiver–adolescent ADHD classifications ranged from approximately zero in a clinically referred inner-city sample to 0.51 in a community epidemiologic cohort, and they changed when impairment or subtype rules changed [36,42,43,44,45]. For example, Jensen et al. [44] reported κ = 0.16 without diagnosis-specific impairment and κ = 0.08 when impairment was required, whereas Rohde et al. [45] reported κ = 0.45 and Cantwell et al. [42] reported κ = 0.51. Hope et al. [46] likewise found limited mother–adolescent diagnostic concordance (39.4%; κ = 0.30), with mothers reporting more ADHD symptoms than adolescents (*M* = 9.42 vs. 8.34, *p* < 0.05). Using independent mother and adolescent K-SADS-E interviews, Gau et al. [47] found small-to-moderate agreement at adolescent follow-up (ICC = 0.32–0.56), compared with higher agreement at the corresponding childhood baseline assessment (ICC = 0.44–0.71). These values should not be treated as direct equivalents because prevalence, reference rules, interview procedures and case definitions differed.

### 3.5. Caregiver–Teacher Agreement

**Figure 4:**
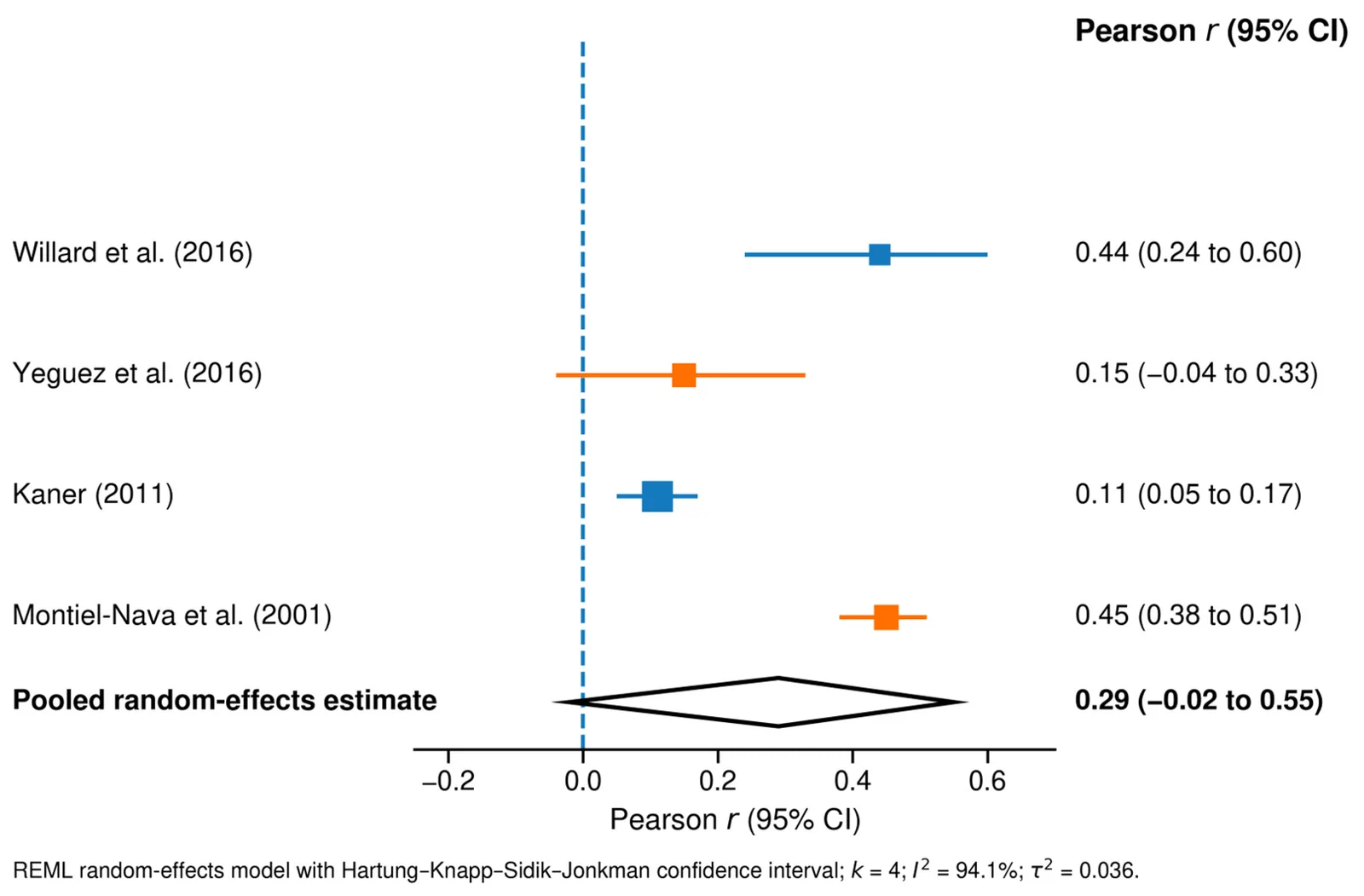
Forest plot of caregiver–teacher agreement for inattention [3,37,39,41]. Note: Individual confidence intervals were calculated by normal approximation on the Fisher *z* scale; the pooled confidence interval used the Hartung–Knapp–Sidik–Jonkman method. Effect sizes and confidence intervals were back-transformed to Pearson *r* for presentation.

**Figure 5:**
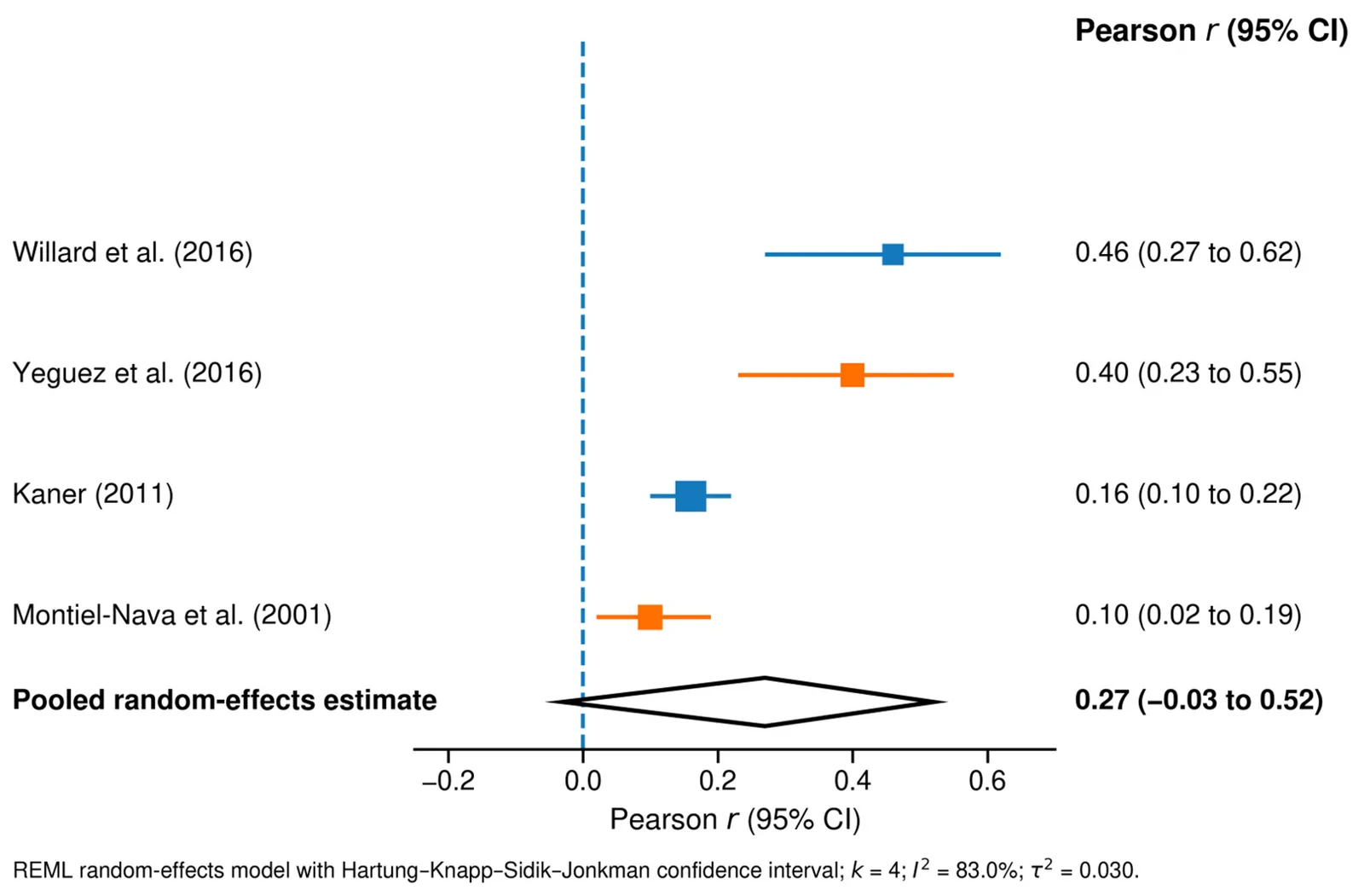
Forest plot of caregiver–teacher agreement for hyperactivity/impulsivity [3,37,39,41]. Note: Individual confidence intervals were calculated by normal approximation on the Fisher *z* scale; the pooled confidence interval used the Hartung–Knapp–Sidik–Jonkman method. Effect sizes and confidence intervals were back-transformed to Pearson *r* for presentation.

Four studies contributed to each caregiver–teacher model [3,37,39,41]. The pooled point estimate for inattention was *r* = 0.29, but its HKSJ interval included zero (95% CI −0.02–0.55, *p* = 0.060). Heterogeneity was high, *Q*(3) = 51.16, *p* < 0.001, *I*^2^ = 94.1%, τ^2^ = 0.036. The hyperactivity/impulsivity estimate was *r* = 0.27 (95% CI −0.03–0.52, *p* = 0.064), with *Q*(3) = 17.65, *p* < 0.001, *I*^2^ = 83.0% and τ^2^ = 0.030. Prediction intervals were not calculated because only four studies contributed to each model. With only four studies per model and *I*^2^ values of 94.1% and 83.0%, these estimates should be interpreted as highly uncertain average effects rather than stable expected levels of caregiver–teacher agreement. This caution is reinforced by the methodological quality analysis: after exclusion of High-concern reports, only two studies remained in each model, which was insufficient for a meaningful quality-restricted meta-analysis. The caregiver–teacher pooled estimates should therefore be regarded as more tentative than the caregiver–adolescent pooled estimates. Study-level estimates are shown in Figure 4 and Figure 5.

Additional parent–teacher evidence used metrics that were not compatible with the Pearson-correlation models. Juneja et al. [48] reported paired parent and teacher Conners classifications in 500 adolescents aged 10–15 years; the reported margins and overlap yielded exact agreement of 0.94 and a derived Cohen’s κ of 0.66. Wan Salwina et al. [49] reported weak parent–teacher correspondence in 12-year-old adolescents, including coefficients of 0.107 for inattention, 0.241 for hyperactivity, and 0.093 for combined symptoms. These coefficients were retained narratively because the report described the statistic inconsistently as an intraclass correlation and a Spearman correlation. Frick et al. [50] used parent and teacher DSM symptom ratings in a latent-class model at age 10 and identified both pervasive and setting-specific ADHD patterns, including Pervasive Combined (10.5%), School Combined (9.2%), School Inattentive (15.3%), and Home Combined (18.2%) classes.

Other parent–teacher reports used kappa, ICC, latent correlations or systematic discrepancy scores. The direction and magnitude of agreement varied by developmental stratum, instrument, reporter and definition. Correlation-based results sometimes indicated low-to-moderate rank-order consistency while categorical agreement remained low, reinforcing the need to keep dimensional and threshold results separate [51,52].

### 3.6. Teacher–Adolescent, Teacher–Teacher and Clinician Comparisons

Teacher–adolescent evidence was too heterogeneous for pooling. Gadow et al. [40] reported that all youth–teacher ADHD symptom correlations were below 0.18, whereas Wan Salwina et al. [49] reported weak or near-zero adolescent–teacher coefficients across hyperactive, inattentive, and combined symptom domains. Studies used Pearson correlations, ICCs, model-based shared-trait variance, signed-rank tests and broader attention-problem measures, often with different age restrictions. In an adolescent twin subgroup, Martin et al. [53] found higher self-reported hyperactivity scores than both parent ratings (*Z* = −3.102, *p* = 0.002) and teacher ratings (*Z* = −7.244, *p* < 0.001). The overall pattern suggested lower and less consistent convergence across school and self-report contexts than within more similar settings, but the available estimates could not be reduced to a single valid coefficient [35,54,55,56].

Teacher–teacher agreement varied according to the teacher pair, instrument and whether consistency or absolute agreement was estimated. Morris et al. [57] reported average-measures absolute-agreement ICCs of 0.57–0.71 across Math and English teachers in two adolescent cohorts. Molina et al. [58] reported dimensional ICCs around 0.35–0.51 and categorical kappas of 0.17–0.40 across instruments. Danforth and DuPaul [59] reported stronger correlations in an older subgroup, but the exact subgroup size and age cutoff were unavailable.

Comparisons with clinicians or consensus diagnoses also required cautious interpretation. A clinician rating was not an independent reference standard when it incorporated the same adolescent or parent report under evaluation. Joint interviews, fixed informant order and consensus procedures could either increase apparent agreement or redistribute discrepancies [42,45,60].

### 3.7. Sensitivity Analyses

For caregiver–adolescent inattention, exclusion of High-concern reports reduced the pooled estimate to *r* = 0.24 (*k* = 4, 95% CI 0.06–0.40) and *I*^2^ to 33.1%. For hyperactivity/impulsivity, the corresponding estimate was *r* = 0.27 (*k* = 4, 95% CI −0.03–0.52), with residual heterogeneity of *I*^2^ = 74.8%. Alternative-measure analyses remained positive but heterogeneous: *r* = 0.37 (95% CI 0.20–0.53; *I*^2^ = 90.7%) for inattention and *r* = 0.30 (95% CI 0.16–0.43; *I*^2^ = 84.0%) for hyperactivity/impulsivity. In leave-one-out analyses, omission of Kaner [37] reduced *I*^2^ from 88.1% to 46.9% for inattention and from 94.8% to 68.7% for hyperactivity/impulsivity. Omission of the other reports generally left heterogeneity high or very high. Because Kaner [37] differed simultaneously in sample size, setting, instrument, and methodological quality, these influence results do not identify a single causal moderator.

The caregiver–teacher quality-restricted models could not be estimated because only two studies remained. Alternative-measure estimates were *r* = 0.32 (95% CI 0.08–0.53; *I*^2^ = 87.4%) for inattention and *r* = 0.24 (95% CI −0.10–0.54; *I*^2^ = 87.3%) for hyperactivity/impulsivity. Across leave-one-out analyses, all intervals crossed zero and *I*^2^ remained between 78.5% and 96.0%, indicating that no single report accounted for the heterogeneity. Fixed-effect estimates were more precise but were retained only as sensitivity analyses because they do not represent between-study variation. The random-effects HKSJ results remained primary. Complete results are reported in Table S6.

### 3.8. Integrated Synthesis

Across all evidence streams, informant reports were positively related on average but were not clinically interchangeable. Agreement depended on the pair of observers, setting, assessment format, symptom domain and case definition. Dimensional correlations did not guarantee diagnostic agreement, and diagnostic agreement could change after adding impairment or consensus rules. Formal subgroup analyses and meta-regression were not performed. Each primary model contained only four or seven studies, and no candidate moderator was available in a sufficiently consistent form across the contributing reports. Age ranges were broad and overlapping. Table S3 reports the eligible age range or stratum and mean age where available. Across the seven caregiver–adolescent primary studies, mean ages ranged from 12.78 to 16.24 years, but the contributing age ranges overlapped substantially (11–18 years overall). Among the four caregiver–teacher studies, mean age was available for three and ranged from 12.78 to 16.24 years; the remaining report provided separate 12–14 and 15–17-year strata. These data permit descriptive inspection of developmental coverage but do not support a defensible test of an age gradient. Sex distribution was not available for all studies in a form that could be harmonized, and parental ADHD, family characteristics, medication status, school context, and recruitment setting were either unavailable or defined differently. Study-specific moderator analyses also used different outcomes and interaction specifications. Any post hoc subgrouping would therefore have produced very small, non-comparable groups and unstable estimates. The available moderator findings were retained narratively. Rothen et al. [61] found no clear evidence that diagnostic agreement differed between adolescents aged 13–17 years and children aged 7–12 years (OR = 1.46, 95% CI 0.76–2.83). In the adolescent interview subgroup examined by Faraone et al. [62], mothers consistently reported more ADHD symptoms than youths, but the magnitude of this discrepancy did not differ according to maternal or paternal ADHD status (interaction *F* = 0.50, *p* = 0.61). The most defensible overall conclusion is therefore not that one informant is generally more accurate, but that each source contributes a partly overlapping and partly context-bound account. The integrated evidence streams are summarized in Table 2.

## 4. Discussion

### 4.1. Principal Findings

This systematic review provides an adolescence-specific synthesis of cross-informant agreement in ADHD assessment across dimensional symptom ratings, diagnostic classifications and several informant pairings. Caregiver–adolescent agreement was positive but modest for both core domains, with pooled correlations of 0.33 for inattention and 0.32 for hyperactivity/impulsivity. Caregiver–teacher estimates were of a similar magnitude, although only four studies contributed to each model and the Hartung–Knapp–Sidik–Jonkman confidence intervals included zero. These findings are consistent with the broader multi-informant literature, which indicates that reports from different observers usually share meaningful variance but rarely provide clinically interchangeable descriptions of child or adolescent psychopathology [7,8,9,24]. These pooled correlations should be interpreted as average effects across heterogeneous studies rather than as stable expected levels of agreement.

The similarity of the pooled estimates for inattention and hyperactivity/impulsivity is noteworthy. Hyperactive or impulsive behaviors might be expected to be more directly observable than difficulties involving sustained attention, organization or mental effort. However, greater behavioral visibility does not necessarily produce greater agreement when the behavior is expressed differently across settings or when informants apply different thresholds for judging it as developmentally inappropriate. The present results do not support the conclusion that either core ADHD domain is consistently less vulnerable to informant disagreement. Both domains showed very high heterogeneity, and the prediction intervals ranged from negative to strong positive values. Thus, although the average association was positive, the evidence does not support a single expected magnitude of caregiver–adolescent agreement across clinical, educational, or cultural settings.

The caregiver–teacher estimates require particularly cautious interpretation. Heterogeneity was also high in these models (*I*^2^ = 94.1% and 83.0%), and prediction intervals could not be estimated because only four studies contributed to each analysis. Their confidence intervals included zero under the prespecified random-effects model, but this should not be interpreted as evidence that reports from home and school are unrelated. The point estimates were comparable with those observed for caregiver–adolescent agreement and with low-to-moderate associations reported in previous developmental studies [2,31,51,52]. The imprecision mainly reflects the small number of contributing studies and the marked between-study variation. Methodological quality further weakens confidence in these estimates: exclusion of High-concern reports left only two studies per domain, preventing estimation of a quality-restricted pooled effect. The caregiver–teacher estimates therefore have a weaker evidential basis than the caregiver–adolescent pooled estimates and should not be interpreted as having comparable precision or robustness. Fixed-effect analyses produced more precise estimates because large studies received dominant weight, but such models do not adequately represent variation across populations, instruments and school contexts. The random-effects findings therefore provide a more conservative account of the available evidence.

Diagnostic and threshold-based findings strengthened the conclusion that informant reports cannot be treated as substitutes. Chance-corrected agreement was frequently low, and estimates changed when impairment requirements, subtype rules or consensus procedures were modified [42,43,44,45]. This apparent difference between dimensional and categorical findings is not contradictory. A correlation describes whether informants rank adolescents in a similar order, whereas diagnostic agreement depends on whether both reports cross a specified threshold. Even a relatively small difference near that threshold may change the resulting classification. Diagnostic concordance is consequently influenced not only by the similarity of the underlying ratings but also by symptom prevalence, the placement of the threshold, the requirement for impairment and the rule used to integrate information [23,24,25,26,27,28,29].

### 4.2. Relation to Previous Literature

The present findings extend rather than contradict earlier work on multi-informant assessment. Broad reviews of child and adolescent psychopathology have repeatedly shown that informant discrepancies are common and cannot be explained solely as measurement error [7,8,9]. The multi-informant approach is valuable precisely because observers encounter the young person under different conditions and may provide information about different portions of the same clinical problem [8,17]. Our results demonstrate that this principle remains highly relevant when the analysis is restricted to ADHD during adolescence, a developmental period in which observation opportunities and the adolescent’s role in assessment change substantially.

Previous reviews have addressed related but distinct questions. Staff et al. [31] examined the validity of teacher rating scales against interviews and classroom observations, whereas Garcia-Rosales et al. [30] reviewed whether ADHD symptom criteria function equivalently across parent and teacher ratings. The current review synthesized observed agreement and discrepancy between informants; it did not test measurement invariance. These evidence streams are complementary but should not be conflated. A teacher scale may show acceptable validity against an external criterion while agreeing only modestly with a parent rating. Conversely, evidence of measurement non-invariance would indicate that items or latent dimensions function differently across reporters, but such non-invariance cannot be inferred from a modest correlation or a score difference alone. The present findings therefore support the clinical non-interchangeability of informant reports, while psychometric equivalence must be evaluated through dedicated invariance or related analyses.

Adolescence also distinguishes the present evidence from much of the earlier child-focused literature. As adolescents spend more time outside direct parental supervision, caregivers may have less access to school behavior, peer interactions and unstructured activities. At the same time, secondary-school teachers often observe an adolescent for only one subject or a limited part of the week. Adolescents have access to effort, distractibility and subjective impairment that may not be evident to adults, but they may have less opportunity for comparison with same-age peers or may interpret symptoms differently from parents and teachers [11,12,13,14]. Studies of adolescent self-report have accordingly shown that it is neither wholly unreliable nor closely concordant with adult reports [34,35,36,37,38,39,40,60]. The modest pooled correlations observed here are compatible with the view that adolescent self-report contains clinically relevant information while remaining incomplete when considered in isolation.

### 4.3. Why Agreement Varies

Contextual specificity is one likely contributor to the high heterogeneity. ADHD symptoms are not expressed independently of environmental demands. A structured classroom, an examination, an unstructured peer setting and a familiar home routine place different demands on sustained attention, inhibition, organization and emotional regulation. Symptoms may also vary within the same adolescent across school and non-school days and across different parts of the day [21]. Divergent ratings may therefore reflect real variation in the conditions under which difficulties emerge rather than an error that must be attributed to one informant [8,9,17].

Observation opportunities also differ substantially. Parents usually observe behavior over longer periods and across family routines, but may have little direct knowledge of classroom functioning. Teachers can compare an adolescent with many same-age peers, although the selected teacher, subject and classroom structure can materially affect the resulting assessment [3,31,51]. Evidence from studies involving more than one teacher confirms that agreement varies according to the teacher pair, instrument and statistical definition employed [57,58,59]. In secondary education, a single teacher report should not automatically be assumed to represent functioning throughout the entire school environment.

Informants also differ in their frames of reference. A caregiver may judge behavior against the adolescent’s earlier functioning or the behavior of siblings. A teacher is more likely to compare the adolescent with classmates and to emphasize conduct that interferes with classroom management or academic completion. Adolescents may focus on internal effort, distress or situations that adults do not observe. Expectations about age-appropriate behavior, family relationships, school climate and sociocultural context may further influence the threshold at which a behavior is regarded as problematic [8,16,17,18,19]. The resulting discrepancy may therefore reveal whose functioning is affected and under which conditions, even when it does not identify a single objectively correct score.

Parent–adolescent relationship processes may also influence caregiver–adolescent agreement. Open communication is particularly relevant because caregivers can only report some aspects of an adolescent’s experience to the extent that these experiences are observable or disclosed. Conversely, adolescents who experience communication with a parent as less open may be less likely to discuss difficulties involving attention, effort, organization or impairment that occur outside direct parental observation. Recent cross-sectional evidence indicates that adolescents with ADHD who were not receiving pharmacological treatment reported lower open communication with both mothers and fathers than typically developing adolescents after adjustment for age and problematic internet use, whereas group differences were not consistently observed across broader positive and negative relationship dimensions [63]. Adolescents receiving pharmacological treatment did not differ significantly from controls in open communication in that sample [63]. This study did not examine cross-informant ADHD agreement directly and therefore cannot establish that communication explains informant discrepancies. Rather, it identifies parent–adolescent communication as a plausible relational factor that future agreement studies should test explicitly.

Methodological variation is another important explanation. Parallel rating scales improve comparability, but informants may still receive differently worded items, use different reference periods or complete forms with different normative frameworks. Measurement invariance across parent and teacher ADHD ratings cannot be assumed and must be tested directly; the agreement estimates synthesized here do not themselves establish non-invariance [30]. Interviews allow clarification and explicit assessment of impairment but can introduce interviewer, order and information-contamination effects. In several diagnostic studies, the clinician or consensus classification incorporated information from one or both informants being evaluated, meaning that it could not serve as an entirely independent reference standard [42,44,45,60].

The sensitivity analyses did not isolate a single explanation for the observed heterogeneity. Omission of Kaner [37] reduced *I*^2^ to 46.9% for caregiver–adolescent inattention and to 68.7% for hyperactivity/impulsivity, but this report differed simultaneously in sample size, national setting, instrument, and methodological quality. In the caregiver–teacher analyses, heterogeneity remained high after every single-study omission. These patterns suggest that several overlapping population and design differences may contribute to the variation, but the small number of studies prevents their independent effects from being separated.

The choice of agreement statistic also shaped the evidence. Pearson correlations measure rank-order consistency but cannot detect a systematic tendency for one informant to assign higher scores. Intraclass correlations depend on whether consistency or absolute agreement is specified, while kappa coefficients depend on the distribution of categorical decisions. Mean differences can demonstrate directional bias without showing correspondence between individuals. Latent multi-trait multi-method models can separate shared trait variance from informant-specific variance, but their parameters do not translate directly into clinical classification agreement [34,54]. Studies reporting more than one of these indices provided a more informative description than studies that labeled a single correlation as “agreement.”

### 4.4. Clinical Implications

The findings support retaining source-specific information throughout adolescent ADHD assessment. Diagnostic guidelines require evidence concerning symptoms and impairment across settings, but they do not require parents, adolescents and teachers to provide identical ratings [1,10]. Clinicians should initially record each report separately, including the setting observed, duration of observation, medication status, functional demands and any opportunity for informants to influence one another. Integration should occur after these contextual features have been examined, rather than by immediately averaging scores or privileging one source as the default truth.

When ratings diverge, the next clinical task is to determine the structure of the discrepancy. Item-level review may show that informants agree about some behaviors but differ about others. Follow-up questions should examine whether symptoms occur only under particular academic, social or family demands; whether one informant has had limited opportunity to observe the behavior; whether the disagreement concerns symptom presence or functional impact; and whether scale items were interpreted in the same way. This approach is consistent with models that treat informant discrepancies as potentially meaningful data about the young person’s context rather than merely as an obstacle to diagnosis [8,17,18].

Adolescent self-report should be included but interpreted within this broader framework. Modest agreement with caregivers does not justify dismissing the adolescent’s account. Adolescents can describe the effort needed to maintain concentration, subjective restlessness, compensatory strategies, experiences outside adult observation and the personal consequences of symptoms [11,12,13,14]. Conversely, self-report alone may not capture longitudinal change, comparative deviation from same-age peers or impairment visible to others. The most defensible interpretation therefore combines the adolescent’s perspective with external observations while preserving areas of disagreement [36,38,39,60].

Teacher information remains particularly important because ADHD diagnostic formulation requires evidence beyond the home environment [1,10]. Nevertheless, the findings argue against treating any single teacher as a complete representation of school functioning. When feasible, information should be obtained from teachers who know the adolescent well and across more than one subject or classroom context. When substantial discrepancies remain after differences in setting, observation opportunities, timing, medication status, and item interpretation have been considered, additional information should be sought from sources that are as independent as possible of the discrepant ratings. Relevant evidence may include attendance and assignment-completion records, changes in academic functioning, disciplinary information, reports from another teacher or school professional, and, when feasible, direct observation. These sources should not be used to “vote” between informants or to create a mechanical average of their reports. Instead, they should be mapped to the specific symptoms, settings, and functional consequences described by each informant to determine where independent corroboration exists. If clinically important discrepancies remain unresolved, that uncertainty should be retained explicitly in the formulation, and diagnostic or treatment decisions should be based on the overall pattern of symptoms, impairment, developmental history, and cross-setting evidence rather than on forced agreement between reporters [1,10,31,51,57,58].

Integration rules should also be matched to the clinical purpose. Requiring agreement between all informants may miss difficulties that are severe but setting-specific, whereas classifying an adolescent whenever any informant endorses a symptom may increase the number of positive classifications. Averaging informant scores can predict broader impairment in some circumstances, but may conceal clinically meaningful contrasts between home, school and self-report [2,24]. No single mathematical rule removes the need for clinical evaluation of symptom duration, developmental appropriateness, cross-setting expression and impairment [1,10].

### 4.5. Implications for Research

Future studies should report adolescent results separately from those of younger children. Twenty-nine otherwise relevant full-text reports were excluded because findings for participants aged 10–19 years could not be separated. This loss of evidence could be avoided by presenting age-stratified coefficients or by depositing sufficiently detailed analytic data. Age should not be treated only as a covariate in broad child samples, because the transition into adolescence changes school organization, autonomy, parental observation and the role of self-report. In the present evidence base, age was also confounded with study-level differences in instrument, setting, sampling and informant composition; the descriptive age information in Table S3 should therefore not be interpreted as evidence for or against a developmental effect on agreement.

A minimum reporting set for cross-informant studies should include the analysis sample for every estimate, the exact symptom domain and instrument, the informant pair, confidence intervals, administration timing, medication condition, independence of completion and the statistical definition of agreement. For categorical outcomes, complete cross-tabulations and the classification rule should be provided. For parallel continuous scales, correlations should be accompanied by information about systematic mean differences and, where appropriate, absolute-agreement ICCs. These procedures would address several of the recurrent methodological concerns identified through the QAREL appraisal [33].

Studies should also distinguish between consistency, absolute agreement, diagnostic concordance and agreement about impairment. These are separate questions and may have different clinical implications. Before interpreting score differences, investigators should examine whether the instrument functions comparably across informants [30]. When clinician or consensus decisions are used as comparators, reports should specify exactly which informant data were available to the decision makers so that incorporation of the index report into the reference classification can be recognized.

The evidence base did not permit reliable conclusions about whether disagreement is moderated by age, sex, parental ADHD, family characteristics, school context or cultural background. A further candidate moderator, highlighted by recent relational research but not directly tested in the agreement studies synthesized here, is the quality of parent–adolescent communication [63]. Future caregiver–adolescent agreement studies should therefore assess open communication and broader relationship quality directly, preferably separately for mothers and fathers, and test whether these factors account for variation in symptom agreement. Medication status and problematic internet use may also warrant consideration as related contextual variables, while avoiding causal interpretation unless supported by longitudinal or experimental designs [63]. Future moderator analyses should be prespecified and use comparable definitions. Large longitudinal studies would be particularly informative because they could distinguish developmental change in agreement from changes caused by the transition to multiple secondary-school teachers or reduced parental observation. Repeated parallel assessments could also determine whether particular discrepancy patterns predict later impairment, treatment engagement or diagnostic stability rather than merely describing cross-sectional disagreement [2,3,20,51].

### 4.6. Strengths and Limitations

This review has several strengths. It addressed a clearly defined adolescence-specific question, searched medical, nursing, educational and multidisciplinary databases, and included comparisons involving adolescents, caregivers, teachers and clinicians. Extraction was conducted at result level, preserving distinctions between symptom domain, informant pair, instrument, age stratum and diagnostic rule. Correlations were not combined with kappas, ICCs or agreement proportions, because these statistics quantify different properties. The use of restricted-maximum-likelihood random-effects models and Hartung–Knapp–Sidik–Jonkman intervals avoided giving unwarranted precision to meta-analyses containing only a small number of studies. The review also documented four reports that could not be retrieved in full rather than silently omitting them from the study selection record.

Several limitations should be considered. The original title-and-abstract screening, data extraction, and methodological quality appraisal were performed by one reviewer. During peer-review revision, a second reviewer independently rescreened all 796 unique records, reassessed all 64 retrieved full-text reports, verified every study-domain effect entering the four final primary meta-analyses, and independently repeated the adapted QAREL appraisal of all included reports. These procedures substantially strengthen confidence in the final evidence set and primary quantitative analyses, but they do not fully replace a prospectively implemented duplicate workflow. In particular, the complete set of 282 result-level rows used across the narrative and quantitative evidence streams was not independently extracted in duplicate.

Registration was not fully prospective. Database searching and initial retrieval preceded registration, so the search process and eligibility framework already used to identify records were not constrained by the registered protocol. Registration did, however, precede formal extraction, methodological quality appraisal, effect grouping, and quantitative synthesis, and the revision now distinguishes registered plans from subsequent operational specifications. A chronology of methodological decisions relative to registration is provided in Table S7.

APA PsycInfo was not available through the accessible institutional interface. Although the search included Scopus and Academic Search Complete to broaden coverage beyond biomedical databases, no separate formal citation-chasing stage was undertaken. Psychology-focused studies indexed preferentially in PsycInfo, as well as grey literature reports, may therefore have been missed. This limits confidence that the search captured the complete relevant evidence base. Although no language restriction was imposed and one Spanish-language report was included, the final evidence base was overwhelmingly English-language (29 of 30 reports). Thus, the geographical spread of the included studies across 13 countries or national settings should not be interpreted as equivalent linguistic or cultural coverage.

Four potentially eligible reports could not be retrieved in full, and each meta-analysis contained only four or seven studies. Formal subgroup analysis or meta-regression would therefore have produced very small groups or unstable coefficients, particularly because candidate moderators were incompletely and inconsistently reported. The absence of formal moderator analysis should not be interpreted as evidence that age, sex, parental ADHD, family characteristics, medication status, or school context are unimportant. Rather, the very high and largely unexplained heterogeneity means that the summary correlations are average effects across diverse studies, not universal constants for adolescent ADHD assessment. The wide confidence and prediction intervals should be given greater weight than the numerical similarity of the pooled point estimates.

The methodological quality of the primary evidence also limits the precision of the conclusions. Only three of the 30 included reports were judged Low concern, whereas 15 had Some concerns and 12 were judged High concern. Recurrent problems included incomplete reporting of independent completion and masking, fixed assessment order or access to another informant’s information, nonparallel instruments, age-stratum uncertainty, and use of correlation alone to characterize agreement. These issues may either inflate apparent correspondence through information contamination or make estimates difficult to interpret because the informants, instruments, or statistical definitions are not fully comparable.

The quality-restricted sensitivity analyses illustrate the practical importance of these concerns. Excluding High-concern reports reduced the caregiver–adolescent pooled estimate to *r* = 0.24 for inattention, with *I*^2^ = 33.1%, and to *r* = 0.27 for hyperactivity/impulsivity, with *I*^2^ = 74.8%. For the caregiver–teacher models, exclusion of High-concern reports left only two studies, precluding a meaningful quality-restricted meta-analysis. The numerical pooled correlations should therefore be interpreted cautiously, particularly for caregiver–teacher agreement. Greater confidence can be placed in the broader conclusion that reports from different informants provide partly overlapping but non-interchangeable information than in any single pooled estimate as a precise expected level of agreement.

Finally, the review did not apply GRADE certainty ratings. QAREL was useful for identifying methodological problems in individual reliability studies, but its judgments do not provide a complete assessment of certainty across a heterogeneous evidence stream [33]. The conclusions have therefore been framed cautiously: the evidence indicates that informant reports are related but not clinically interchangeable, whereas the exact magnitude of agreement and the effects of particular moderators remain uncertain. Psychometric equivalence across informant versions was not directly assessed in this review.

## 5. Conclusions

Cross-informant agreement in adolescent ADHD assessment was positive on average but highly heterogeneous and imprecisely estimated. The caregiver–adolescent prediction intervals crossed zero, and the caregiver–teacher confidence intervals also included zero; the pooled correlations should therefore be interpreted as average effects across diverse studies rather than stable levels expected in a particular setting. Caregiver, adolescent, teacher, and clinician reports should be treated as related but not clinically interchangeable sources of evidence; this conclusion does not establish psychometric non-equivalence of the instruments. The clinically useful response to disagreement is not to select a universally superior informant, but to investigate what each source observed and how the discrepancy relates to context and impairment. Better age-stratified reporting and harmonized moderator data are needed before narrower conclusions can be made about sources of heterogeneity or specific assessment procedures.

## Figures and Tables

**Table 1:** Primary random-effects meta-analyses of cross-informant Pearson correlations.

Informant Pair	Domain	*k*	Pooled *r*	95% CI	*p*	*I* ^2^	τ^2^	95% Prediction Interval
Caregiver–adolescent	Inattention	7	0.33	0.19–0.46	0.001	88.1%	0.022	−0.07–0.64
Caregiver–adolescent	Hyperactivity/impulsivity	7	0.32	0.12–0.50	0.009	94.8%	0.050	−0.28–0.74
Caregiver–teacher	Inattention	4	0.29	−0.02–0.55	0.060	94.1%	0.036	Not calculated (*k* < 5)
Caregiver–teacher	Hyperactivity/impulsivity	4	0.27	−0.03–0.52	0.064	83.0%	0.030	Not calculated (*k* < 5)

Note: Correlations were pooled on the Fisher *z* scale using restricted maximum likelihood and back-transformed to Pearson *r*. Pooled confidence intervals use the Hartung–Knapp–Sidik–Jonkman method. Prediction intervals were calculated only when *k* ≥ 5. Because heterogeneity was high, the pooled correlations should be interpreted as average effects across studies rather than expected values for a specific population or setting.

**Table 2:** Structured narrative synthesis of evidence streams not represented fully by the primary meta-analyses.

Evidence Stream	Outcome Scope	Synthesis Finding	Confidence
Caregiver–adolescent dimensional	Core inattention and hyperactivity/impulsivity	Average pooled *r* = 0.32–0.33; very high heterogeneity (*I*^2^ = 88.1–94.8%); prediction intervals ranged from −0.28 to 0.74 across the two core-domain models and crossed zero.	Very cautious
Caregiver–teacher dimensional	Core inattention and hyperactivity/impulsivity	Point estimates *r* = 0.27–0.29; HKSJ confidence intervals included zero; high heterogeneity (*I*^2^ = 83.0–94.1%); four studies per model; quality-restricted models could not be estimated because only two studies remained after exclusion of High-concern reports.	Very cautious
Caregiver–adolescent diagnostic	Attention-deficit/hyperactivity disorder diagnoses, subtype and impairment thresholds	Generally limited chance-corrected agreement; estimates changed with impairment and consensus rules.	Very cautious
Teacher–adolescent	Core symptoms and broader attention scales	Direction generally positive, but metrics and constructs were incompatible for pooling.	Very cautious
Teacher–teacher	Dimensional and categorical school ratings	Agreement depended on teacher pair, instrument, subgroup and reliability definition.	Very cautious
Clinician or consensus	Symptoms and diagnostic classifications	Interpretation was limited when the reference decision incorporated the informant under comparison.	Very cautious
Moderators	Age, sex, parental attention-deficit/hyperactivity disorder, family and school characteristics	Study-specific findings were too inconsistently defined and analyzed for pooled inference.	Exploratory

Note: Confidence labels are descriptive judgments about interpretive caution and are not GRADE certainty ratings.

## Data Availability

No new primary data were generated. The OSF registration and the study selection/full-text screening workbook are available in the associated Open Science Framework project (https://doi.org/10.17605/OSF.IO/49H5R (accessed on 20 August 2026)). Study characteristics, methodological quality judgments, primary meta-analytic inputs, and sensitivity analyses are reported in the Supplementary Materials (Tables S3–S6). Copyrighted full-text reports will not be redistributed. Additional review materials are available from the corresponding author upon reasonable request.

## Supplementary Materials

The following supporting information can be downloaded at https://www.mdpi.com/article/10.3390/children13091176/s1, Table S1. PRISMA 2020 checklist [32]. Table S2. Database search strategies and yields. Table S3. Characteristics of included reports. Table S4. Methodological quality appraisal of included reports using the adapted QAREL framework. Table S5. Study-level inputs for the primary meta-analyses. Table S6. Sensitivity and robustness analyses. Table S7. Timing and status of methodological decisions relative to OSF registration.

## Abbreviations

The following abbreviations are used in this manuscript:

ADHD	Attention-deficit/hyperactivity disorder
CI	Confidence interval
DSM	Diagnostic and Statistical Manual of Mental Disorders
H/I	Hyperactivity/impulsivity
HKSJ	Hartung–Knapp–Sidik–Jonkman
ICC	Intraclass correlation coefficient
OSF	Open Science Framework
PRISMA	Preferred Reporting Items for Systematic Reviews and Meta-Analyses
QAREL	Quality Appraisal of Reliability Studies
REML	Restricted maximum likelihood
