# Supplementary material for: Cross-Informant Agreement and Discrepancies in ADHD Assessment During Adolescence: A Systematic Review and Meta-Analysis
Source: Children (Basel). 2026 Sep 1;13(9):1176. doi: 10.3390/children13091176 (PMC13605905; doi:10.3390/children13091176)
Supplement: Supplementary file 1 [file children-13-01176-s001.zip › Table S2.pdf]

Supplementary Table S2. Database search strategies and yields.

Database	Interface	Search date	Query string	Records
MEDLINE	EBSCO	19 July 2026	(ADHD OR "attention deficit hyperactivity disorder" OR "attention-deficit/hyperactivity disorder" OR "hyperkinetic disorder") AND (adolescen* OR teen*) AND ("cross-informant*" OR "multi-informant*" OR "informant agreement" OR "informant disagreement" OR "informant discrepanc*" OR "rater agreement" OR "rater disagreement" OR "parent-teacher agreement" OR "parent teacher agreement" OR "parent-adolescent agreement" OR "parent adolescent agreement" OR "teacher-adolescent agreement" OR "teacher adolescent agreement" OR "self-report discrepanc*" OR "positive illusory bias" OR "positive illusion*")	243
CINAHL with Full Text	EBSCO	19 July 2026	(ADHD OR "attention deficit hyperactivity disorder" OR "attention-deficit/hyperactivity disorder" OR "hyperkinetic disorder") AND (adolescen* OR teen*) AND ("cross-informant*" OR "multi-informant*" OR "informant agreement" OR "informant disagreement" OR "informant discrepanc*" OR "rater agreement" OR "rater disagreement" OR "parent-teacher agreement" OR "parent teacher agreement" OR "parent-adolescent agreement" OR "parent adolescent agreement" OR "teacher-adolescent agreement" OR "teacher adolescent agreement" OR "self-report discrepanc*" OR "positive illusory bias" OR "positive illusion*")	75
ERIC	EBSCO	19 July 2026	(ADHD OR "attention deficit hyperactivity disorder" OR "attention-deficit/hyperactivity disorder" OR "hyperkinetic disorder") AND (adolescen* OR teen*) AND ("cross-informant*" OR "multi-informant*" OR "informant agreement" OR "informant disagreement" OR "informant discrepanc*" OR "rater agreement" OR "rater disagreement" OR "parent-teacher agreement" OR "parent teacher agreement" OR "parent-adolescent agreement" OR "parent adolescent agreement" OR "teacher-adolescent agreement" OR "teacher adolescent agreement" OR "self-report discrepanc*" OR "positive illusory bias" OR "positive illusion*")	29
Academic Search Complete	EBSCO	19 July 2026	(ADHD OR "attention deficit hyperactivity disorder" OR "attention-deficit/hyperactivity disorder" OR "hyperkinetic disorder") AND (adolescen* OR teen*) AND ("cross-informant*" OR "multi-informant*" OR "informant agreement" OR "informant disagreement" OR "informant discrepanc*" OR "rater agreement" OR "rater disagreement" OR "parent-teacher agreement" OR "parent teacher agreement" OR "parent-adolescent agreement" OR "parent adolescent agreement" OR "teacher-adolescent agreement" OR "teacher adolescent agreement" OR "self-report discrepanc*" OR "positive illusory bias" OR "positive illusion*")	165

Supplementary Table S2. Database search strategies and yields (continued).

Database	Interface	Search date	Query string	Records
PubMed	PubMed	19 July 2026	(ADHD[tiab] OR "attention deficit hyperactivity disorder"[tiab] OR "attention-deficit/hyperactivity disorder"[tiab] OR "hyperkinetic disorder"[tiab] OR "Attention Deficit Disorder with Hyperactivity"[Mesh]) AND (adolescen*[tiab] OR teen*[tiab] OR youth*[tiab] OR "Adolescent"[Mesh]) AND (informant*[tiab] OR rater*[tiab] OR reporter*[tiab] OR parent*[tiab] OR caregiver*[tiab] OR teacher*[tiab] OR clinician*[tiab] OR self-report*[tiab]) AND (agreement[tiab] OR concordan*[tiab] OR discrepanc*[tiab] OR reliability[tiab] OR correlation*[tiab] OR kappa[tiab] OR "intraclass correlation"[tiab] OR "positive illusory bias"[tiab] OR "positive illusion*"[tiab])	531
Scopus	Scopus	19 July 2026	TITLE-ABS-KEY((ADHD OR "attention deficit hyperactivity disorder" OR "attention-deficit/hyperactivity disorder" OR "hyperkinetic disorder") AND (adolescen* OR teen* OR youth*) AND (informant* OR rater* OR reporter* OR parent* OR caregiver* OR teacher* OR clinician* OR "self-report*") AND (agreement OR concordan* OR discrepanc* OR reliability OR correlation* OR kappa OR "intraclass correlation" OR "positive illusory bias" OR "positive illusion*"))	404
Gross total	—	—	—	1,447

Note. APA PsycInfo was not available through the accessible institutional interface and was not searched directly. Coverage was broadened through the inclusion of Scopus and Academic Search Complete alongside MEDLINE, PubMed, CINAHL and ERIC. No separate formal backward- or forward-citation-chasing stage was conducted. Platform-specific syntax and field tags were adapted as shown; no publication-year or language limits were applied.
