# Supplementary material for: Cross-Informant Agreement and Discrepancies in ADHD Assessment During Adolescence: A Systematic Review and Meta-Analysis
Source: Children (Basel). 2026 Sep 1;13(9):1176. doi: 10.3390/children13091176 (PMC13605905; doi:10.3390/children13091176)
Supplement: Supplementary file 1 [file children-13-01176-s001.zip › Table S3.pdf]

Supplementary Table S3. Characteristics of included reports.

Report	Country	*n*	Eligible age range/stratum (years)	Mean age (SD), years	Informants	Primary instrument/method	QAREL
Frick et al. [60]	Sweden	159	15–18	16.59 (0.92)	Adolescent and parent compared with clinician	ASRS-A plus MINI-KID ADHD section	High concern
Frick et al. [50]	United Kingdom	10,476 overall; age-10 item data 7,576–8,018	Age-10 assessment	NR	Parent–teacher	Parent and teacher ratings of 18 DSM ADHD symptoms; latent class analysis	Some concerns
Morris et al. [57]	United States	335 (agreement *n* = 304)	13–15.9, two cohorts	14.25 (0.88); 13.96 (1.17)	Math teacher–English teacher	SNAP-IV Teacher Rating Scale	Some concerns
Sibley et al. [38]	United States	107	11–15	12.78 (1.70)	Caregiver–adolescent	DSM-5 ADHD Rating Scale	Some concerns
Hennig et al. [35]	Germany	114	12–17	14.00 (1.50)	Parent, teacher, and adolescent; pairwise and three-rater comparisons	DISYPS-KJ ADHD questionnaires	Some concerns
Willard et al. [39]	United States	80	12–17	15.00 (1.84)	Parent, teacher, and adolescent; pairwise dyads	Conners Rating Scale, 3rd edition (Conners 3)	Some concerns
Yeguez and Sibley [3]	United States	112	11–15	12.78 (0.86)	Mother–teacher	Disruptive Behavior Disorders Rating Scale (DBD)	Some concerns
Gomez and Gomez [54]	Australia	214	12–17	13.82 (1.30)	Adolescent–mother or teacher	Disruptive Behavior Rating Scale (DBRS)	Low concern
Narad et al. [51]	United States	1,689	11–13.99 and 14–17.99 strata	NR	Parent–teacher	Vanderbilt ADHD Diagnostic Parent and Teacher Rating Scales (VADPRS/VADTRS)	Some concerns
Hogue et al. [36]	United States	168	12–18	15.10 (1.30)	Caregiver–adolescent	Mini International Neuropsychiatric Interview (MINI), Version 5.0	Some concerns
Juneja et al. [48]	India	500	10–15	NR; median 13	Parent–teacher	Conners Parent and Teacher Rating Scales–Revised, Short Forms; paired ADHD classification	Some concerns

Report	Country	*n*	Eligible age range/stratum (years)	Mean age (SD), years	Informants	Primary instrument/method	QAREL
Wan Salwina et al. [49]	Malaysia	410; parent-involving comparisons n = 367	12 (all participants)	12.0 (all participants aged 12)	Adolescent, parent and teacher	Conners short forms; domain-specific cross-informant coefficients	High concern
Kaner [37]	Turkey	937	12–18	16.24 (0.96)	Parent and teacher compared with adolescent	Conners Revised Long Forms: CPRS-R/L, CTRS-R/L and CASS-L	High concern
Gau et al. [47]	Taiwan	93	11–16	13.20 (1.40)	Mother–adolescent	Chinese K-SADS-E	Low concern
Rothen et al. [61]	Switzerland	296	7–17 overall; adolescent stratum 13–17 in moderator analysis	NR for adolescent stratum	Offspring direct interview compared with mother and/or father family-history report	Modified K-SADS-E and Family History-Research Diagnostic Criteria (FH-RDC)	Some concerns
Colins et al. [43]	Belgium	115	12–17	NR	Caregiver–adolescent	Diagnostic Interview Schedule for Children, Version IV (DISC-IV), parent and youth versions	Some concerns
Serra-Pinheiro et al. [52]	Brazil	205	10–16	12.00 (1.13)	Parent–teacher	MTA SNAP-IV	Some concerns
Pierrehumbert et al. [55]	Switzerland	915	12–17 eligible self-report subgroup	NR	Parent or teacher–adolescent	Conners' Rating Scales–Revised, Short Forms (CPRS-R:S, CTRS-R:S and CASS:S)	High concern
Hartung et al. [34]	United States	203	13–18	15.21 (1.37)	Parent/legal guardian–adolescent	Disruptive Behavior Disorders Checklist (DBD), rating-scale and semistructured-interview formats	High concern
Faraone et al. [62]	United States	156	12–18	NR	Mother–adolescent aged 12 or older	Schedule for Affective Disorders and Schizophrenia for School-Age Children—Epidemiologic Version (K-SADS-E)	Some concerns

Report	Country	*n*	Eligible age range/stratum (years)	Mean age (SD), years	Informants	Primary instrument/method	QAREL
Steinhausen et al. [56]	Switzerland	35	10–16	12.80 (1.30)	Mother via DISC interview compared with teacher via TRF or adolescent via YSR	DISC 2.3 plus CBCL, TRF and YSR cross-informant Attention Problems scales	High concern
Gadow et al. [40]	United States	239; teacher ratings n = 214	11–18	13.90 (1.80)	Adolescent, parent and teacher	YI-4 and parallel parent/teacher ASI-4 forms	High concern
Martin et al. [53]	Wales, United Kingdom	572	11–16 eligible self-report subgroup	NR	Adolescent–parent or teacher	Strengths and Difficulties Questionnaire (SDQ), hyperactivity subscale	High concern
Montiel-Nava and Peña [41]	Venezuela	530	12–14 and 15–17 strata	NR	Parent–teacher	Conners' Rating Scales–Revised, Long Forms for parents and teachers	High concern
Hope et al. [46]	United States	121	11–17	13.17 (1.89)	Mother–adolescent	Diagnostic Interview for Children and Adolescents–Revised, Parent and Adolescent Forms (DICA-R-P/DICA-R-A)	Some concerns
Jensen et al. [44]	United States	840	12–17	NR	Parent/primary caretaker–adolescent	Computerized Diagnostic Interview Schedule for Children, Version 2.3 (PC-DISC)	Low concern
Rohde et al. [45]	Brazil	191	12–14	NR	Parent/primary caregiver–adolescent	Clinician DSM-IV ADHD diagnostic interview using separate parent and adolescent reports	High concern
Molina et al. [58]	United States	88	13–18	15.15 (1.43)	Teacher 1–Teacher 2 or multiple teachers	Teacher Report Form (TRF), IOWA/Abbreviated Conners, and Disruptive Behavior Disorders Rating Scale (DBD)	Some concerns
Cantwell et al. [42]	United States	281	14–18	16.70 (1.20)	Parent–adolescent	K-SADS hybrid (Epidemiologic + Present Episode versions)	High concern

Report	Country	*n*	Eligible age range/stratum (years)	Mean age (SD), years	Informants	Primary instrument/method	QAREL
Danforth and DuPaul [59]	United States	46	Older-than-median stratum of overall 8–17 sample; exact cutoff NR	NR for eligible stratum	Coteacher 1–Coteacher 2	Eight teacher rating instruments for ADHD-related behavior	High concern

Note. Sample sizes and age values refer to the eligible adolescent analysis set or age stratum used in this review where available, rather than necessarily to the full report sample. Mean age (SD) is reported where available for the eligible set; NR, not reported. Juneja et al. [48] reported a median age of 13 years rather than a mean. Narad et al. [51] and Montiel-Nava and Peña [41] contributed separate adolescent age strata. For Rothen et al. [61] and Danforth and DuPaul [59], an adolescent or older-subgroup result was eligible, but the mean age of that specific subgroup was not reported. ADHD = attention-deficit/hyperactivity disorder; QAREL = Quality Appraisal of Reliability Studies. No publication-language restriction was applied. Twenty-nine included reports were published in English and one, Montiel-Nava and Peña [41], was published in Spanish. Caregiver is used as the umbrella term in synthesis-level reporting; source-specific terms such as mother, father, parent, caretaker, or legal guardian are retained when describing individual reports.
