# Supplementary material for: Cross-Informant Agreement and Discrepancies in ADHD Assessment During Adolescence: A Systematic Review and Meta-Analysis
Source: Children (Basel). 2026 Sep 1;13(9):1176. doi: 10.3390/children13091176 (PMC13605905; doi:10.3390/children13091176)
Supplement: Supplementary file 1 [file children-13-01176-s001.zip › Table S4.pdf]

Supplementary Table S4. Methodological quality appraisal of included reports using the adapted QAREL framework.

Report	Q1	Q2	Q3	Q4	Q5	Q6	Q7	Q8	Q9	Q10	Q11	Overall
Frick et al. [60]	Yes	Yes	No	N/A	Unclear	No	Unclear	Unclear	Unclear	Yes	Yes	High concern
Frick et al. [50]	Yes	Yes	Unclear	N/A	N/A	Unclear	Unclear	N/A	Yes	Yes	Yes	Some concerns
Morris et al. [57]	Yes	Yes	Unclear	N/A	N/A	Unclear	Unclear	N/A	Yes	Yes	Yes	Some concerns
Sibley et al. [38]	Yes	Yes	Unclear	N/A	N/A	Unclear	Unclear	N/A	Yes	Yes	Yes	Some concerns
Hennig et al. [35]	Yes	Yes	Unclear	N/A	N/A	Unclear	Unclear	N/A	Yes	Yes	Yes	Some concerns
Willard et al. [39]	Yes	Yes	Unclear	N/A	N/A	Unclear	Unclear	N/A	Yes	Yes	Yes	Some concerns
Yeguez and Sibley [3]	Yes	Yes	Unclear	N/A	N/A	Unclear	Unclear	N/A	Yes	Yes	Yes	Some concerns
Gomez and Gomez [54]	Yes	Yes	Yes	N/A	N/A	Unclear	Unclear	N/A	Yes	Yes	Yes	Low concern
Narad et al. [51]	Yes	Yes	Unclear	N/A	N/A	Unclear	Unclear	N/A	Yes	Yes	Unclear	Some concerns
Hogue et al. [36]	Yes	Yes	Unclear	N/A	N/A	Unclear	Unclear	Unclear	Yes	Yes	Yes	Some concerns
Juneja et al. [48]	Yes	Yes	Unclear	N/A	N/A	Unclear	Unclear	N/A	Yes	Yes	Yes	Some concerns
Wan Salwina et al. [49]	Yes	Yes	Unclear	N/A	N/A	Unclear	Unclear	N/A	Yes	No	No	High concern
Kaner [37]	Unclear	Unclear	Unclear	N/A	N/A	Unclear	Unclear	N/A	Unclear	Yes	No	High concern
Gau et al. [47]	Yes	Yes	Yes	N/A	Yes	Unclear	Yes	Unclear	Yes	Yes	Yes	Low concern
Rothen et al. [61]	Unclear	Yes	Yes	N/A	Yes	Unclear	Unclear	N/A	Yes	Yes	Yes	Some concerns
Colins et al. [43]	Yes	Yes	Unclear	N/A	N/A	Unclear	Unclear	Unclear	Yes	Yes	Yes	Some concerns
Serra-Pinheiro et al. [52]	Yes	Yes	Unclear	N/A	N/A	Unclear	Unclear	N/A	Yes	Yes	Yes	Some concerns
Pierrehumbert et al. [55]	Yes	Yes	Unclear	N/A	N/A	Unclear	Unclear	N/A	Yes	Yes	No	High concern
Hartung et al. [34]	Yes	Yes	Unclear	N/A	N/A	Unclear	Unclear	Yes	Yes	Yes	No	High concern

Report	Q1	Q2	Q3	Q4	Q5	Q6	Q7	Q8	Q9	Q10	Q11	Overall
Faraone et al. [62]	Yes	Yes	Unclear	N/A	N/A	Unclear	Unclear	Unclear	Yes	Yes	Yes	Some concerns
Steinhausen et al. [56]	No	Yes	Unclear	N/A	N/A	Unclear	Unclear	N/A	Yes	Unclear	No	High concern
Gadow et al. [40]	Yes	Yes	Unclear	N/A	N/A	Unclear	Unclear	N/A	Yes	Yes	No	High concern
Martin et al. [53]	Unclear	Yes	Unclear	N/A	N/A	Unclear	Unclear	N/A	Yes	Yes	No	High concern
Montiel-Nava and Peña [41]	Yes	Yes	Unclear	N/A	N/A	Unclear	Unclear	N/A	Yes	Yes	No	High concern
Hope et al. [46]	Yes	Yes	Unclear	N/A	N/A	Unclear	Unclear	Unclear	Yes	Yes	Yes	Some concerns
Jensen et al. [44]	Yes	Yes	Yes	N/A	Unclear	Unclear	Unclear	Unclear	Yes	Yes	Yes	Low concern
Rohde et al. [45]	Yes	Unclear	No	N/A	Unclear	No	Unclear	No	Yes	Yes	Yes	High concern
Molina et al. [58]	Yes	Yes	Unclear	N/A	N/A	Unclear	Unclear	N/A	Yes	No	Yes	Some concerns
Cantwell et al. [42]	Yes	Yes	No	N/A	Unclear	No	Unclear	No	Yes	Yes	Yes	High concern
Danforth and DuPaul [59]	No	Yes	Yes	N/A	N/A	Unclear	Yes	N/A	Yes	Yes	No	High concern

Note. Q1, participant spectrum; Q2, rater spectrum; Q3, masking to the other rater's findings; Q4, masking to the rater's own prior findings; Q5, masking to clinical information; Q6, masking to other cues; Q7, masking to expected prevalence or agreement; Q8, order effects; Q9, time interval; Q10, correct application and interpretation; Q11, appropriate statistical analysis. Cell judgments were Yes, No, Unclear, or N/A. N/A, not applicable; QAREL, Quality Appraisal of Reliability Studies. Overall judgments were qualitative; no summed score was calculated. Across the 30 included reports, 3 were judged Low concern, 15 Some concerns, and 12 High concern. QAREL was adapted operationally for cross-informant ADHD studies. Q1–Q2 addressed participant and informant spectra; Q3–Q8 independence of ratings, masking, access to additional cues, and order effects; Q9 temporal proximity of assessments; Q10 correct application and interpretation of the agreement measure; and Q11 appropriateness of the statistical analysis. Items that were not applicable to a particular assessment design were coded N/A. Overall judgments reflected the likely impact of methodological concerns on interpretation of the agreement estimate and were not derived from a summed score.
