# Supplementary material for: Cross-Informant Agreement and Discrepancies in ADHD Assessment During Adolescence: A Systematic Review and Meta-Analysis
Source: Children (Basel). 2026 Sep 1;13(9):1176. doi: 10.3390/children13091176 (PMC13605905; doi:10.3390/children13091176)
Supplement: Supplementary file 1 [file children-13-01176-s001.zip › Table S5.pdf]

Supplementary Table S5. Study-level inputs for the primary meta-analyses.

Analysis	Study	QAREL	*r*	Analysis *n*	95% CI	Weight	Outcome ID	Selection reason	Source
MA1	Sibley et al. [38]	Some concerns	0.230	107	0.04–0.40	12.9%	ADHD-277-O01	Full-sample baseline correlation for the parallel inattention scale.	*p*. 309
MA1	Hennig et al. [35]	Some concerns	0.380	113	0.21–0.53	13.1%	ADHD-330-O02	Full-sample baseline correlation for the parallel inattention scale.	Table 1, *p*. 83
MA1	Willard et al. [39]	Some concerns	0.210	80	−0.01–0.41	11.6%	ADHD-365-O06	Full-sample correlation for the parallel Conners 3 inattention scale.	Tables 2–3, pp. 1114–1115
MA1	Hogue et al. [36]	Some concerns	0.140	167	−0.01–0.29	14.4%	ADHD-423-O01	Full-sample dimensional MINI inattention/disorganization correlation.	Results, Cross-informant agreement
MA1	Kaner [37]	High concern	0.540	937	0.49–0.58	17.5%	ADHD-496-O09	DSM-IV inattention subscale selected over the broader cognitive problems/inattention scale.	Table 1, pp. 3344–3345
MA1	Hartung et al. [34]	High concern	0.320	203	0.19–0.44	15.0%	ADHD-650-O03	Parallel rating-scale format selected for comparability with the other primary studies; interview-format result retained for sensitivity analysis.	Table V and Results, pp. 166–167
MA1	Gadow et al. [40]	High concern	0.390	239	0.28–0.49	15.4%	ADHD-710-O01	Full-sample correlation for the parallel YI-4/parent ASI-4 inattention domain.	p. 676
MA2	Sibley et al. [38]	Some concerns	0.330	107	0.15–0.49	13.6%	ADHD-277-O02	Full-sample baseline correlation for the parallel H/I scale.	*p*. 309
MA2	Hennig et al. [35]	Some concerns	0.470	113	0.31–0.60	13.7%	ADHD-330-O06	Full-sample baseline correlation for the parallel H/I scale.	Table 1, *p*. 83
MA2	Willard et al. [39]	Some concerns	0.070	80	−0.15–0.29	12.9%	ADHD-365-O08	Full-sample correlation for the parallel Conners 3 H/I scale.	Tables 2–3, pp. 1114–1115
MA2	Hogue et al. [36]	Some concerns	0.160	166	0.01–0.30	14.4%	ADHD-423-O02	Full-sample dimensional MINI H/I correlation.	Results, Cross-informant agreement
MA2	Kaner [37]	High concern	0.620	937	0.58–0.66	15.8%	ADHD-496-O10	DSM-IV H/I subscale selected over the broader hyperactivity scale.	Table 1, pp. 3344–3345
MA2	Hartung et al. [34]	High concern	0.140	203	0.00–0.27	14.7%	ADHD-650-O04	Parallel rating-scale format selected for comparability with the other primary studies; interview-format result retained for sensitivity analysis.	Table V and Results, pp. 166–167

Analysis	Study	QAREL	*r*	Analysis *n*	95% CI	Weight	Outcome ID	Selection reason	Source
MA2	Gadow et al. [40]	High concern	0.320	239	0.20–0.43	14.9%	ADHD-710-O02	Full-sample correlation for the parallel YI-4/parent ASI-4 hyperactivity/impulsivity domain.	p. 676
MA3	Willard et al. [39]	Some concerns	0.440	80	0.24–0.60	21.3%	ADHD-365-O02	Full-sample correlation for the parallel Conners 3 inattention scale.	Tables 2–3, pp. 1114–1115
MA3	Yeguez and Sibley [3]	Some concerns	0.150	112	−0.04–0.33	23.1%	ADHD-384-O01	Full-sample baseline correlation for parallel DBD inattention scores.	Table 2 and Results
MA3	Kaner [37]	High concern	0.110	937	0.05–0.17	28.1%	ADHD-496-O03	DSM-IV inattention subscale selected over the broader cognitive problems/inattention scale.	Table 1, pp. 3344–3345
MA3	Montiel-Nava and Peña [41]	High concern	0.448	527	0.38–0.51	27.5%	ADHD-724-O03; ADHD-724-O08	Non-overlapping age 12–14 and 15–17 DSM-IV inattention strata combined within study on the Fisher-*z* scale.	Table IV, *p*. 508; Table V, *p*. 509
MA4	Willard et al. [39]	Some concerns	0.460	80	0.27–0.62	20.7%	ADHD-365-O04	Full-sample correlation for the parallel Conners 3 H/I scale.	Tables 2–3, pp. 1114–1115
MA4	Yeguez and Sibley [3]	Some concerns	0.400	112	0.23–0.55	22.7%	ADHD-384-O03	Full-sample baseline correlation for parallel DBD H/I scores.	Table 2 and Results
MA4	Kaner [37]	High concern	0.160	937	0.10–0.22	28.7%	ADHD-496-O04	DSM-IV H/I subscale selected over the broader hyperactivity scale.	Table 1, pp. 3344–3345
MA4	Montiel-Nava and Peña [41]	High concern	0.102	527	0.02–0.19	27.9%	ADHD-724-O04; ADHD-724-O09	Non-overlapping age 12–14 and 15–17 DSM-IV H/I strata combined within study on the Fisher-*z* scale.	Table IV, *p*. 508; Table V, *p*. 509

Note. MA1, caregiver–adolescent agreement for inattention; MA2, caregiver–adolescent agreement for hyperactivity/impulsivity; MA3, caregiver–teacher agreement for inattention; MA4, caregiver–teacher agreement for hyperactivity/impulsivity. Analysis *n* denotes the sample contributing to the specific effect and may differ from the report-level sample presented in Table S3 because of outcome-specific missing data or the combination of non-overlapping age strata. For Montiel-Nava and Peña [41], the effective analysis *n* of 527 reflects the combination on the Fisher *z* scale of two non-overlapping strata with $n = 243$ and $n = 287$. Study-specific 95% confidence intervals were calculated by normal approximation on the Fisher *z* scale and back-transformed to Pearson's *r*. Random-effects weights are analysis-specific and may not sum to exactly 100% because of rounding. CI, confidence interval; DBD, Disruptive Behavior Disorders Rating Scale; DSM-IV, Diagnostic and Statistical Manual of Mental Disorders, Fourth Edition; H/I, hyperactivity/impulsivity; MINI, Mini International Neuropsychiatric Interview; QAREL, Quality Appraisal of Reliability Studies.
