# Supplementary material for: Cross-Informant Agreement and Discrepancies in ADHD Assessment During Adolescence: A Systematic Review and Meta-Analysis
Source: Children (Basel). 2026 Sep 1;13(9):1176. doi: 10.3390/children13091176 (PMC13605905; doi:10.3390/children13091176)
Supplement: Supplementary file 1 [file children-13-01176-s001.zip › Table S6.pdf]

Supplementary Table S6. Sensitivity and robustness analyses.

Analysis	Check	Detail	*k*	Pooled *r*	95% CI	*p*	τ^2	I^2
MA1	Fixed-effect comparison	Inverse-variance fixed-effect model on Fisher *z*	7	0.43	0.39–0.47	<0.001	—	88.1%
MA1	Exclusion of High-concern reports	Kaner [37], Hartung et al. [34], and Gadow et al. [40] excluded	4	0.24	0.06–0.40	0.024	0.005	33.1%
MA1	Alternative measure/format	Kaner [37] cognitive problems/inattention; Hartung et al. [34] interview; Gadow et al. [40] retained	7	0.37	0.20–0.53	0.002	0.037	90.7%
MA1	Leave-one-out	Omitted Sibley et al. [38]	6	0.35	0.18–0.49	0.003	0.024	88.8%
MA1	Leave-one-out	Omitted Hennig et al. [35]	6	0.32	0.15–0.48	0.005	0.027	90.0%
MA1	Leave-one-out	Omitted Willard et al. [39]	6	0.35	0.19–0.49	0.003	0.024	89.0%
MA1	Leave-one-out	Omitted Hogue et al. [36]	6	0.37	0.23–0.49	0.001	0.018	84.5%
MA1	Leave-one-out	Omitted Kaner [37]	6	0.29	0.18–0.39	0.001	0.006	46.9%
MA1	Leave-one-out	Omitted Hartung et al. [34]	6	0.33	0.16–0.49	0.005	0.027	89.3%
MA1	Leave-one-out	Omitted Gadow et al. [40]	6	0.32	0.15–0.47	0.005	0.027	90.0%

Supplementary Table S6. Sensitivity and robustness analyses (continued: MA2).

Analysis	Check	Detail	k	Pooled r	95% CI	p	τ^2	I^2
MA2	Fixed-effect comparison	Inverse-variance fixed-effect model on Fisher z	7	0.46	0.42–0.50	<0.001	—	94.8%
MA2	Exclusion of High-concern reports	Kaner [37], Hartung et al. [34], and Gadow et al. [40] excluded	4	0.27	−0.03–0.52	0.067	0.028	74.8%
MA2	Alternative measure/format	Kaner [37] broader hyperactivity; Hartung et al. [34] interview; Gadow et al. [40] retained	7	0.30	0.16–0.43	0.003	0.021	84.0%
MA2	Leave-one-out	Omitted Sibley et al. [38]	6	0.32	0.06–0.54	0.025	0.061	95.6%
MA2	Leave-one-out	Omitted Hennig et al. [35]	6	0.30	0.05–0.51	0.027	0.055	95.7%
MA2	Leave-one-out	Omitted Willard et al. [39]	6	0.36	0.13–0.54	0.010	0.048	95.0%
MA2	Leave-one-out	Omitted Hogue et al. [36]	6	0.35	0.11–0.55	0.015	0.054	94.7%
MA2	Leave-one-out	Omitted Kaner [37]	6	0.25	0.09–0.40	0.010	0.017	68.7%
MA2	Leave-one-out	Omitted Hartung et al. [34]	6	0.35	0.11–0.55	0.013	0.052	94.2%
MA2	Leave-one-out	Omitted Gadow et al. [40]	6	0.32	0.06–0.54	0.025	0.061	95.4%

Supplementary Table S6. Sensitivity and robustness analyses (continued: MA3–MA4).

Analysis	Check	Detail	*k*	Pooled *r*	95% CI	*p*	τ^2	I^2
MA3	Fixed-effect comparison	Inverse-variance fixed-effect model on Fisher *z*	4	0.24	0.20–0.29	<0.001	—	94.1%
MA3	Exclusion of High-concern reports	Not estimated because only two studies remained	2	—	—	—	—	—
MA3	Alternative measure/format	Use Kaner [37] cognitive problems/inattention correlation	4	0.32	0.08–0.53	0.025	0.021	87.4%
MA3	Leave-one-out	Omitted Willard et al. [39]	3	0.25	−0.26–0.64	0.169	0.040	95.8%
MA3	Leave-one-out	Omitted Yeguez and Sibley [3]	3	0.33	−0.19–0.71	0.109	0.044	96.0%
MA3	Leave-one-out	Omitted Kaner [37]	3	0.36	−0.09–0.68	0.074	0.027	80.0%
MA3	Leave-one-out	Omitted Montiel-Nava and Peña [41]	3	0.22	−0.24–0.60	0.177	0.028	78.6%
MA4	Fixed-effect comparison	Inverse-variance fixed-effect model on Fisher *z*	4	0.17	0.13–0.22	<0.001	—	83.0%
MA4	Exclusion of High-concern reports	Not estimated because only two studies remained	2	—	—	—	—	—
MA4	Alternative measure/format	Use Kaner [37] broader hyperactivity and Montiel-Nava and Peña [41] H/I factor correlations	4	0.24	−0.10–0.54	0.110	0.043	87.3%
MA4	Leave-one-out	Omitted Willard et al. [39]	3	0.21	−0.19–0.54	0.151	0.020	78.5%
MA4	Leave-one-out	Omitted Yeguez and Sibley [3]	3	0.23	−0.26–0.62	0.184	0.032	80.9%
MA4	Leave-one-out	Omitted Kaner [37]	3	0.31	−0.21–0.70	0.120	0.041	88.4%
MA4	Leave-one-out	Omitted Montiel-Nava and Peña [41]	3	0.33	−0.11–0.66	0.084	0.029	85.2%

Note. MA1, caregiver–adolescent agreement for inattention; MA2, caregiver–adolescent agreement for hyperactivity/impulsivity; MA3, caregiver–teacher agreement for inattention; MA4, caregiver–teacher agreement for hyperactivity/impulsivity. Random-effects sensitivity models used restricted maximum likelihood with untruncated Hartung–Knapp–Sidik–Jonkman confidence intervals; fixed-effect comparisons used inverse-variance weighting. The between-study variance (τ^2) is reported on the Fisher *z* scale and is not applicable to fixed-effect models. Quality-restricted MA3 and MA4 models were not estimated because only two studies remained. Leave-one-out rows identify the report omitted from each model. CI, confidence interval; H/I, hyperactivity/impulsivity; I^2, percentage of total variability attributable to heterogeneity; *k*, number of independent studies; QAREL, Quality Appraisal of Reliability Studies; *r*, pooled Pearson correlation. Changes in I^2 after individual-study omission were treated as influence diagnostics and not as evidence for a specific moderator.
