# Supplementary material for: Cross-Informant Agreement and Discrepancies in ADHD Assessment During Adolescence: A Systematic Review and Meta-Analysis
Source: Children (Basel). 2026 Sep 1;13(9):1176. doi: 10.3390/children13091176 (PMC13605905; doi:10.3390/children13091176)
Supplement: Supplementary file 1 [file children-13-01176-s001.zip › Table S7.pdf]

Supplementary Table S7. Timing and status of methodological decisions relative to OSF registration.

Methodological element	Timing/status	Detail
Review question, age range, broad eligibility framework, information sources, search strings, and search date	Established before registration	Database searches were completed on 19 July 2026; registration occurred on 22 July 2026.
Formal extraction variables and source-location recording	Registered before formal extraction	Extraction included study, sample, informant, instrument, outcome, uncertainty, analysis sample, time point, and source-location information.
Separation of agreement metrics	Registered before synthesis	Pearson correlations, ICCs, kappas, agreement proportions, mean discrepancies, and model-based estimates were retained as distinct evidence streams.
Minimum pooling threshold	Registered before synthesis	At least three independent studies with a compatible metric were required.
Random-effects framework and robustness checks	Registered before synthesis	Random-effects synthesis and sensitivity analyses were specified.
Four domain-specific Pearson models designated as primary quantitative syntheses	Post-registration operational decision	Designation occurred after extraction/effect grouping but before interpretation of pooled results.
Preference for DSM/core-domain scales	Post-registration operational specification	Applied before interpretation of pooled results.
Parallel rating-scale effect selected when multiple formats were available	Post-registration operational specification	Alternative eligible formats were retained for sensitivity analysis.
Exact estimator and interval method	Post-registration statistical specification	REML and untruncated HKSJ confidence intervals were used.
Combination of non-overlapping age strata	Post-registration implementation	Eligible strata from one report were combined on the Fisher-z scale before pooling.
Complete independent rescreening	Peer-review revision	A second reviewer rescreened all 796 unique records and independently reassessed all 64 retrieved reports.

**Methodological element**	**Timing/status**	**Detail**
Evidence-set amendment	Peer-review revision	Four additional eligible reports were included; final evidence set = 30 reports.
Primary-effect and QAREL verification	Peer-review revision	All 22 primary study-domain entries were independently verified and all 30 included reports were independently reappraised with QAREL.
